# *Leptospira interrogans* causes quantitative and morphological disturbances in adherens junctions and other biological groups of proteins in human endothelial cells

**DOI:** 10.1371/journal.pntd.0005830

**Published:** 2017-07-27

**Authors:** Hiromi Sato, Jenifer Coburn

**Affiliations:** Center for Infectious Disease Research, Department of Medicine, Division of Infectious Diseases, Medical College of Wisconsin, Milwaukee, WI, United States of America; Yale University Yale School of Public Health, UNITED STATES

## Abstract

Pathogenic *Leptospira* transmits from animals to humans, causing the zoonotic life-threatening infection called leptospirosis. This infection is reported worldwide with higher risk in tropical regions. Symptoms of leptospirosis range from mild illness to severe illness such as liver damage, kidney failure, respiratory distress, meningitis, and fatal hemorrhagic disease. Invasive species of *Leptospira* rapidly disseminate to multiple tissues where this bacterium damages host endothelial cells, increasing vascular permeability. Despite the burden in humans and animals, the pathogenic mechanisms of *Leptospira* infection remain to be elucidated. The pathogenic leptospires adhere to endothelial cells and permeabilize endothelial barriers *in vivo* and *in vitro*. In this study, human endothelial cells were infected with the pathogenic *L*. *interrogans* serovar Copenhageni or the saprophyte *L*. *biflexa* serovar Patoc to investigate morphological changes and other distinctive phenotypes of host cell proteins by fluorescence microscopy. Among those analyzed, 17 proteins from five biological classes demonstrated distinctive phenotypes in morphology and/or signal intensity upon infection with *Leptospira*. The affected biological groups include: 1) extracellular matrix, 2) intercellular adhesion molecules and cell surface receptors, 3) intracellular proteins, 4) cell-cell junction proteins, and 5) a cytoskeletal protein. Infection with the pathogenic strain most profoundly disturbed the biological structures of adherens junctions (VE-cadherin and catenins) and actin filaments. Our data illuminate morphological disruptions and reduced signals of cell-cell junction proteins and filamentous actin in *L*. *interrogans-*infected endothelial cells. In addition, *Leptospira* infection, regardless of pathogenic status, influenced other host proteins belonging to multiple biological classes. Our data suggest that this zoonotic agent may damage endothelial cells *via* multiple cascades or pathways including endothelial barrier damage and inflammation, potentially leading to vascular hyperpermeability and severe illness *in vivo*. This work provides new insights into the pathophysiological mechanisms of *Leptospira* infection.

## Author summary

Pathogenic *Leptospira* causes the life-threatening infection called leptospirosis worldwide. Symptoms of leptospirosis range from mild illness to severe illness such as organ damage, meningitis, and fatal hemorrhagic disease. Invasive species of *Leptospira* spread to multiple tissues, and damage the linings of blood vessels. Despite the burden in humans and animals, how *Leptospira* bacteria cause damage remains to be determined. In this study, human endothelial cells were infected with *L*. *interrogans* (pathogen) or *L*. *biflexa* (non-pathogen) to investigate changes to host cell proteins. Among those analyzed, 17 proteins from five biological classes demonstrated distinctive changes upon infection with *Leptospira*. Infection with the pathogenic strain most profoundly disturbed the adherens junction group of proteins that hold neighboring cells together. In addition to the changes in cell-cell junctions, *Leptospira* infection, regardless of pathogenic status, influenced other host proteins belonging to multiple biological classes. Our data suggest that *L*. *interrogans* may damage endothelial cells *via* multiple cascades or pathways. The damage may include endothelial barrier disruption and inflammation, potentially leading to leaky blood vessels in patients. This work contributes to our understanding of how of *Leptospira* causes widespread, disseminated infection and disease in humans and animals.

## Introduction

The causative agents of leptospirosis, *Leptospira* species, are Gram-negative spirochetes of the class *Spirochaetales*, along with *Borrelia* and *Treponema* [1]. The genus *Leptospira* has at least 22 species with 300 serovars and are classified as pathogenic, saprophytic, and intermediate types [2–4]. Pathogenic *Leptospira* transmission to humans and susceptible animals causes the zoonotic infection leptospirosis. This life-threatening infection is reported in temperate and especially tropical regions worldwide [5, 6]. The reservoirs of these bacteria are rodents and other domestic and wild animals, which release bacteria-containing urine into water, mud, and soil. Humans exposed to these contaminated sources can be infected through damaged skin or through mucous membranes, including the conjunctiva [1, 2, 6].

The global burden of leptospirosis is estimated to be more than 1 million cases and nearly 60,000 deaths annually [5]. Symptoms are often non-specific but may include high fever, severe headache, chills, myalgia, rash, vomiting, jaundice, red eyes, abdominal pain, and diarrhea [1, 6–8]. Invasive species of *Leptospira* rapidly disseminate to multiple tissues where they damage host endothelial cells and increase vascular permeability, causing more severe illness such as acute renal injury, aseptic meningitis, liver failure, and respiratory distress from acute lung injury [8–12]. Due to the variety of symptoms, patients can be misdiagnosed as having other common viral or bacterial infectious diseases [5, 6, 8]. Leptospirosis can lead to multiple organ failure or fatal hemorrhagic diseases [1, 8–10].

Leptospires are known to adhere to fibroblasts, renal epithelial cells, macrophages, and endothelial cells *in vitro* [13–17]. Multiple *Leptospira* adhesins have been reported to bind cells *via* VE-cadherin or the extracellular matrix (ECM) molecules fibronectin, collagen, laminin, elastin, and plasminogen [18–22]. Binding to glycosaminoglycans (GAGs) may promote attachment to cells and to ECM [18, 19, 22]. The adhesion of pathogenic leptospires is likely an important early stage of the infectious process.

Pathological characteristics of leptospirosis are vasculitis and endothelial cell damage, leading to inflammatory infiltrates, localized ischemia and hemorrhage in organs [1, 2, 11, 12]. Despite the burden in humans and animals, the pathogenic mechanisms of *Leptospira* infection at the cellular and molecular levels are poorly understood. It has been demonstrated that the pathogenic *L*. *interrogans* or its proteins adhere to endothelial cells and permeabilize endothelial cell monolayers *in vitro* [23, 24].

At the molecular level, genetic tools for *Leptospira* work less efficiently than for many other bacteria [25], making studies of *Leptospira* biology challenging. Even when *Leptospira* mutants are constructed, there are few available efficient methods to elucidate the pathogenic mechanisms [26]. Most often, the mutants have been examined for attenuated phenotypes in the mortality of animals or altered histological perturbations of organs from infected animals, such as hamsters [26–30], guinea pigs [30, 31], transgenic mice [32, 33], and zebrafish [34, 35]. Because wild-type mice and rats are carrier animals for leptospires, these animals are used as negative controls to study pathogenicity, e.g. to examine leptospire colonization without disease [26, 36].

As *in vitro* assays to screen *Leptospira* strains, cell attachment and transmigration through polarized epithelial cells have been used [16, 37]. The epithelial translocation of leptospires does not alter the transepithelial electrical resistance [37], so the resistance measurement itself is not informative. Human umbilical vein endothelial cells (HUVEC) are also used to test recombinant leptospiral proteins for changes in host protein expression or cell-junction permeability [24, 38, 39]. To study *Leptospira* pathogenicity more intensely, other *in vitro* screening systems remain to be explored.

In this study, morphological changes and other distinctive phenotypes in *Leptospira*-infected human endothelial cells were investigated. Antibodies and reagents recognizing human proteins were screened by fluorescence microscopy. Most proteins analyzed demonstrated little change in *Leptospira*-infected endothelial cells. Yet, 17 host proteins from five biological classes demonstrated distinctive phenotypes in the morphology and/or signal intensity upon infection: 1) extracellular matrix, 2) intercellular adhesion molecules and cell surface receptors, 3) intracellular proteins, 4) cell-cell junction proteins, and 5) a cytoskeletal protein. The most prominent phenotype of pathogenic *L*. *interrogans* sv. Copenhageni-infected cells was the loss of the adherens junction proteins VE-cadherin and p120-, alpha-, and beta-catenins from the original site at intercellular junctions. Copenhageni infection also influenced the actin cytoskeleton as well as a tight junction protein, ZO-1. Infection with both pathogenic and non-pathogenic *Leptospira* strains altered other host proteins belong to multiple biological classes, although the pathogenic strain caused more intense changes. This work provides the insights in biological and pathological effects of *Leptospira* infection.

## Methods

### *Leptospira* strains and growth conditions

*L*. *interrogans* serovar Copenhageni strain Fiocruz L1-130 (pathogen), *L*. *interrogans* sv. Canicola strain Moulton (pathogen), and *L*. *biflexa* sv. Patoc strain Patoc 1 (non-pathogen) were purchased from ATCC. Bacteria were grown at 30°C in Ellinghausen-McCullough-Johnson-Harris (EMJH) medium supplemented with 1% rabbit serum [40, 41]. The viability, motility, and general morphology of strains were periodically checked using darkfield microscopy. When bacterial cultures reached 1 to 2 × 10^8^ cells/ml, bacteria were used for infection or subcultured in fresh medium. The bacterial cell number was determined using a Petroff-Houser chamber under darkfield microscopy prior to infection. Bacterial cultures of 8 passages or less were used for all experiments. All procedures involving *Leptospira* were performed in a biosafety cabinet.

### *Borrelia burgdorferi* growth conditions

In this study, the wild-type *B*. *burgdorferi* B31-A3 strain was used as a control bacterium. *B*. *burgdorferi* was grown in Barbour-Stoenner-Kelly (BSKII) medium [42] at 33°C to a density of 1 × 10^8^ cells/ml. Prior to each infection, the presence of genomic plasmids was confirmed in each culture by PCR [43, 44].

### Human endothelial cells and growth conditions

The dermal endothelial cell line human microvascular endothelial cells (HMEC-1), was originally a gift from Dr. E. Ades and Dr. T. J. Lawley (Emory School of Medicine and the Centers for Disease Control and Prevention). This cell line is currently available from ATCC. HMEC-1 were cultured in MCDB 131 (Gibco) supplemented with 10 mM L-glutamine, 10 ng/ml epidermal growth factor (Corning), 1 μg/ml hydrocortisone (Sigma), 15% Hyclone Fetal bovine serum (FBS, Thermo), and 25 mM HEPES (Gibco). Two types of primary cells, human dermal lymphatic endothelial cells (HDLEC) and human dermal microvascular endothelial cells (HDMEC), were purchased from ScienCell. These primary cells were cultured in endothelial cell medium (ECM) with the endothelial cell growth supplement (ECGS) and 5% FBS (all from ScienCell), according to the vendor’s protocol. All human cells were grown at 36.5°C under 5% CO_2_. For infection, passage of endothelial cells was limited to 20 or less for HMEC-1 and 13 or less for HDLEC and HDMEC. In preliminary experiments, we confirmed the morphological similarity of HDMEC to the cell line HMEC-1. For screening, HMEC-1 was selected for use as a stable microvascular endothelial cell type, and for some experiments was compared to HDLEC, which possesses morphologically well-organized intercellular junctions.

### Infection of endothelial cells with *Leptospira* strains

Human endothelial cells were grown on sterile coverslips placed in wells of 12-well plates. Seeding numbers used are 4.2 x 10^5^/well for HMEC-1 and 2.2 x 10^5^/well for HDLEC for 2-day growth. Cells were checked under a brightfield microscope for confluence and maturation of intercellular junctions prior to infection. Cells were washed with phosphate-buffered saline (PBS) once, placed in cell culture medium (supplemented MCDB 131 or ECM), and infected with *L*. *interrogans* sv. Copenhageni or *L*. *biflexa* sv. Patoc in EMJH (similar volumes of EMJH were added to the uninfected control cells) at a multiplicity of infection (MOI) of 20 for 24 h at 36.5°C in 5% CO_2_. Infection with *B*. *burgdorferi* B31-A3 was performed under the same conditions as for *Leptospira*. Infected endothelial cells were fixed with 2% para-formaldehyde for 15 min and then rinsed with PBS three times prior to immunofluorescence procedures. All procedures were performed in a biosafety cabinet.

### Immunofluorescence microscopy

The infected and then fixed cells were rinsed with PBS and then either directly used for immunofluorescence procedures without permeabilization or treated with 0.1% Triton X-100 in PBS for 15 min for permeabilization, depending on the cell localization of a host protein or the specificity of an antibody (shown in Table 1). The samples were blocked with 3% bovine serum albumin (BSA) in PBS for 1 h and then incubated in a primary antibody diluted in 3% BSA/PBS for 1 h. For this study, antibodies and reagents were titrated and optimized for immunofluorescence microscopy analyses. The primary antibodies for which data are shown and the dilution factor used are listed in Table 1.

**Table 1:** Reagents for immunofluorescence microscopy.

	company	product No.	reactivity	dilution
**Mouse antibody**				
ICAM-1/CD54 (15.2)	Santa Cruz	sc-15.2	Hu, Ms, Rt	1:300
ICAM-1 (P2A4)	Millipore	MAB2146Z	Hu	1:300
fibronectin (FN-15)	Sigma	F7387	Hu, Ms, Ck	1:2,000
cadherin 5 (VE, CD144, clone 75)	BD Biosciences	610252	Hu	1:300
p120 catenin (15D2)	Millipore	05–1567	Hu, Ms, Rt	1:200, Triton
alpha-catenin (1G5)	Thermo	MA1-2000	Hu, Ms	1:100, Triton
beta-catenin (15B8)	Thermo	MA1-301	Hu, Ms, Rt, Nhp	1:200, Triton
alpha-tubulin (DM1A)	Millipore	05–829	Hu, Ms, Rt	1:200, Triton
**Rabbit antibody**				
collagen type IV (Col4)	anitibodies-online	ABIN707396	Hu, Ms, Rt	1:100
type VI collagen	Telios	A112	Hu	1:1,000
laminin	Sigma	L9393	Hu, Rt	1:1,000
decorin	ThermoFisher	PA5-27370	Hu	1:200
ICAM-2 (H-159)	Santa Cruz	sc-7933	Hu, Ms, Rt	1:500
CD36 (H-300)	Santa Cruz	sc-9154	Hu, Ms, Rt	1:100, Triton
VEGF receptor 2	abcam	ab11939-100	Hu, Ms, Rt	1:100, Triton
VEGF	abcam	ab9570-100	Hu, Ms, Rt, Hm	1:100, Triton
Rho A (119)	Santa Cruz	sc-179	Hu, Ms, Rt	1:600, Triton
ILK (integrin-linked kinase)	Upstate	16–261	Hu	1:10, Trion
nectin 2 (EPR6717)	abcam	ab135246	Hu, Ms, Rt	1:100
claudin-5	abcam	ab15106	Hu, Ms	1:100, Triton
occludin	Invitrogen/Thermo	71–1500	Hu, Ms, Rt, Dg	1:100
ZO-1 (zonula occludens)	ZYMED Lab	61–7300		1:400, Triton
connexin 43 mAb	Cell Signaling	3512S	Hu, Ms, Rt, Nhp	1:50, Triton
**Alexa fluor 488-conjugated reagent**				
phalloidin	Invitrogen	A12379	—	1:400, Triton
anti-mouse IgG cross-adsorbed	Invitrogen	A11029	—	1:1,000
anti-rabbit IgG cross-adsorbed	Invitrogen	A11008	—	1:1,000

*Hu: human, Ms: Mouse, Rt: Rat, Nhp: Non-human primate, Ck: Chicken, Dg: Dog, Hm: Hamster

*”Triton” indicates that fixed cells were permeabilized with 0.1% Triton X-100.

Unbound primary antibody was washed away with 3% BSA/PBS three times prior to incubation with either an anti-mouse-IgG or anti-rabbit-IgG antibody conjugated with Alexa Fluor 488 (Molecular Probes) for detection. After 1 h incubation, the unbound secondary antibody was rinsed away with 3% BSA/PBS twice and then with PBS twice. Filamentous actin was labeled with Alexa Fluor 488-conjugated phalloidin (Table 1) for 20 min and washed with 3% BSA/PBS twice and then with PBS twice. Coverslips were mounted on glass slides using ProLong Diamond containing DAPI (Molecular Probes). The mountant was cured in the dark for 12 h or longer before sealing of the coverslips with nail polish.

Fluorescence microscopy images were acquired by a Nikon Eclipse Ti-U inverted microscope equipped with a CoolSNAP ES2 CCD camera (Photometrics) and a multifluorescent Sedat Quad ET filter set (multichroic splitter, Chroma) using the 20× Plan Apo objective lens (N.A. 0.75, Nikon). NIS-Elements software (Nikon) was used for image acquisition, processing, and analysis. Scale bars represent 50 μm.

### Quantification of fluorescence signal intensity and statistical analysis

In each microscopy experiment, at least 3 to 5 images were acquired, and the experiment was independently repeated at least 3 times for each host protein tested. For the quantification of signal intensity, raw images of each host protein in endothelial cells were processed in NIS-Elements software (Nikon) as follows: adjusting the color values of indexed-color pixels, converting to RGB format, selecting the whole field of an image as a region of interest (ROI), and obtaining the number of the mean intensity indicated in the ROI statistics in the software.

Graphs of the quantified signal intensity were created in Microsoft Excel 2016. Error bars indicate standard deviations (SD) from the means. Statistical analysis was performed using two-tailed unpaired t-test in GraphPad Prism version 7.00. The *p*-values are indicated inside or below the graph.

## Results

### *Leptospira*-mediated changes in endothelial cells

Infection with pathogenic *Leptospira* strains and serovars causes vascular leakage in the tissues and organs of the host organism by increasing the permeability of endothelial layers [8–12]. The mechanisms of this disruption of the endothelial barrier at pathological and molecular levels are unclear. In this study, we used two types of cultured human endothelial cells to investigate which host proteins are affected during infection with pathogenic *L*. *interrogans* strains, compared to the non-pathogenic, saprophytic strain, *L*. *biflexa* sv. Patoc.

For primary screening, the endothelial line HMEC-1 was used due to the faster growth rate and stability of this cell line. The phenotypes of interest detected in HMEC-1 were confirmed and further characterized by infection of primary endothelial cells, human dermal lymphatic endothelial cells (HDLEC). Advantages to the use of HDLEC are: 1) cell size is large, 2) cell structure is generally flat without overlapped cell edges, and 3) the well-defined structure of cell-cell junctions. Endothelial cells were infected with leptospires at a multiplicity of infection (MOI) of 20 for 24 h throughout the screening process of candidate proteins (Methods). Leptospires remained motile throughout the 24 h co-incubation. During incubation, we did not observe a hallmark of apoptosis, nuclear condensation or fragmentation in infected endothelial cells (S5 Fig, DAPI). To investigate the effect of *Leptospira* infection on endothelial cells, we screened antibodies and reagents to detect any changes in biological structures of human proteins by immunofluorescence microscopy.

After repeated screening, we found that the signal intensity or overall morphology of most of the host proteins tested were not significantly influenced by infection with either pathogenic or non-pathogenic leptospires. The lack of change could be a result of the irrelevance of the host protein to *Leptospira* infection or could be due to technical issues, such as the epitope position(s) in the tested protein or the antibody specificity for immunofluorescence microscopy. Among the analyzed reagents, we identified that the signal intensity and/or cellular morphology of 17 host proteins were affected by *Leptospira* infection (Table 2). These 17 human proteins, belonging to 5 biological groups, are the focus of this study.

**Table 2:** Overall effect of *Leptospira* infection on host proteins.

	signal intensity	morphology	specificity
**Extracellular matrix**			
collagen type IV (Col4)	slight increase	puncta	Copenhageni > Patoc
decorin	slight increase	puncta	Copenhageni = Patoc
laminin	slight decrease (HDLEC)	rearrangement	Copenhageni > Patoc
**ICAM/cell surface receptor**			
ICAM-1 (CD54)	increase	not observed	Copenhageni >> Patoc
ICAM-2	increase	puncta	Copenhageni > Patoc
CD36	slight increase	puncta	Copenhageni > Patoc
VEGF receptor 2	slight increase	puncta	Copenhageni = Patoc
**Intracellular protein**			
VEGF	slight increase	puncta	Copenhageni > Patoc
Rho A	slight increase	puncta	Copenhageni > Patoc
ILK (integrin-linked kinase)	slight increase	puncta	Copenhageni = Patoc
**Adherens junction**			
VE-cadherin (cadherin 5, CD144)	decrease	not applicable	Copenhageni
p120 catenin	decrease	not applicable	Copenhageni
alpha-catenin	decrease	not applicable	Copenhageni
beta-catenin	decrease	not applicable	Copenhageni
**Tight junction**			
ZO-1 (zonula occludens)	slight decrease	mislocalization	Copenhageni (HDLEC)
**Gap junction**			
connexin 43	decrease	not applicable	Copenhageni (HDLEC)
connexin 43	not observed	mislocalization	Patoc (HDLEC)
**Actin cytoskeleton**			
filamentous actin	decrease	not applicable	Copenhageni (HMEC-1)
filamentous actin	not observed	mislocalization	Copenhageni (HDLEC)

### Extracellular matrix (ECM) proteins–Collagen type IV, decorin, and laminin

**Fig 1:**
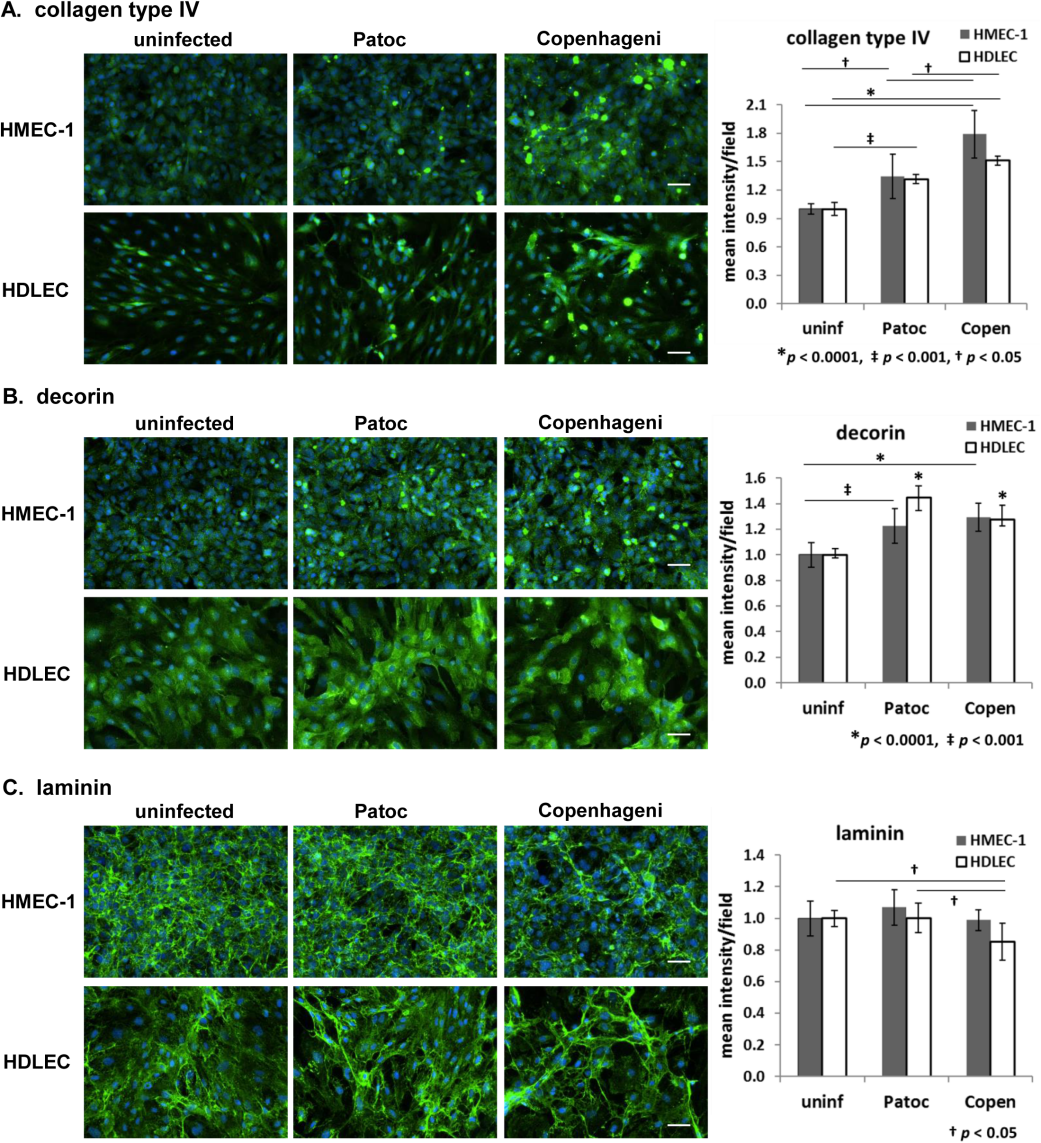
Effect of *Leptospira* infection on extracellular matrix proteins in endothelial cells detected by immunofluorescence microscopy. (A) collagen type IV, (B) decorin, and (C) laminin in HMEC-1 and HDLEC are shown in green. The nuclei are stained in blue for all panels. Scale bars represent 50 μm. Quantified signal intensity of the host protein is indicated in the right-hand graphs (mean +/- SD, *p*-value is indicated below each graph, the independent *p*-values shown as an asterisk are compared to uninfected cells).

We first analyzed several extracellular matrix (ECM) proteins that are known to be the targets of many of the *Leptospira* adhesins [18–21]. In this study, *L*. *interrogans* sv. Copenhageni infection was found to influence three ECM proteins. One is collagen type IV, which is one of the most abundant ECM proteins and is located exclusively in the basement membrane [45]. Collagen type IV provides a scaffold for cell structural stability and also plays a role in interaction of cells with underlying basement membranes, critical for cell adhesion [45]. Compared to uninfected endothelial cells, Copenhageni infection increased the signal intensity by 1.5- to 2-fold with concomitant morphological changes leading to formation of puncta (Fig 1A, Table 2). The signal increase was minor in *L*. *biflexa* sv. Patoc-infected cells, ~1.3-fold (Fig 1A). The signal increase and punctate morphology were also observed with another type of collagen, type VI (S1A Fig).

Decorin is another ECM protein affected by infection. Decorin is a small leucine-rich proteoglycan that associates with fibrillar collagen type I [46, 47]. After *Leptospira* infection, the signal intensity of decorin slightly increased by 1.2- to 1.4-fold, in combination with increases in puncta in both Patoc- and Copenhageni-infected endothelial cells (Fig 1B, Table 2). These data indicate that the changes of decorin signal and morphology are caused by *Leptospira* infection in general, and are not specific to the pathogenic strain.

Laminin is a glycoprotein, a major component of basal lamina located in the basement membrane [48]. In micrographs of uninfected endothelial cells, laminin displayed an intricate net-shaped structure (Fig 1C, uninfected). When cells were infected with Copenhageni, the laminin network appeared to form bundles of small, rolled up, rope-like nets (Fig 1C). There was a minor reduction of the signal intensity in Copenhageni-infected HDLEC but not in HMEC-1 (Fig 1C). In Patoc-infected cells, there were only subtle structural rearrangements or changes in the signal intensity of laminin (Fig 1C). These data suggest that the rearrangement of the laminin structure is a pathogenic Copenhageni-specific phenotype.

Fibronectin is one of most abundant ECM proteins in tissues, along with collagen and laminin. There was no detectable change in fibronectin morphology upon infection with either Copenhageni or Patoc, although the signal intensity of fibronectin was slightly increased (S1B Fig).

### Intercellular adhesion molecules and cell surface receptors–ICAM-1, ICAM-2, CD-36, and VEGF-receptor 2

**Fig 2:**
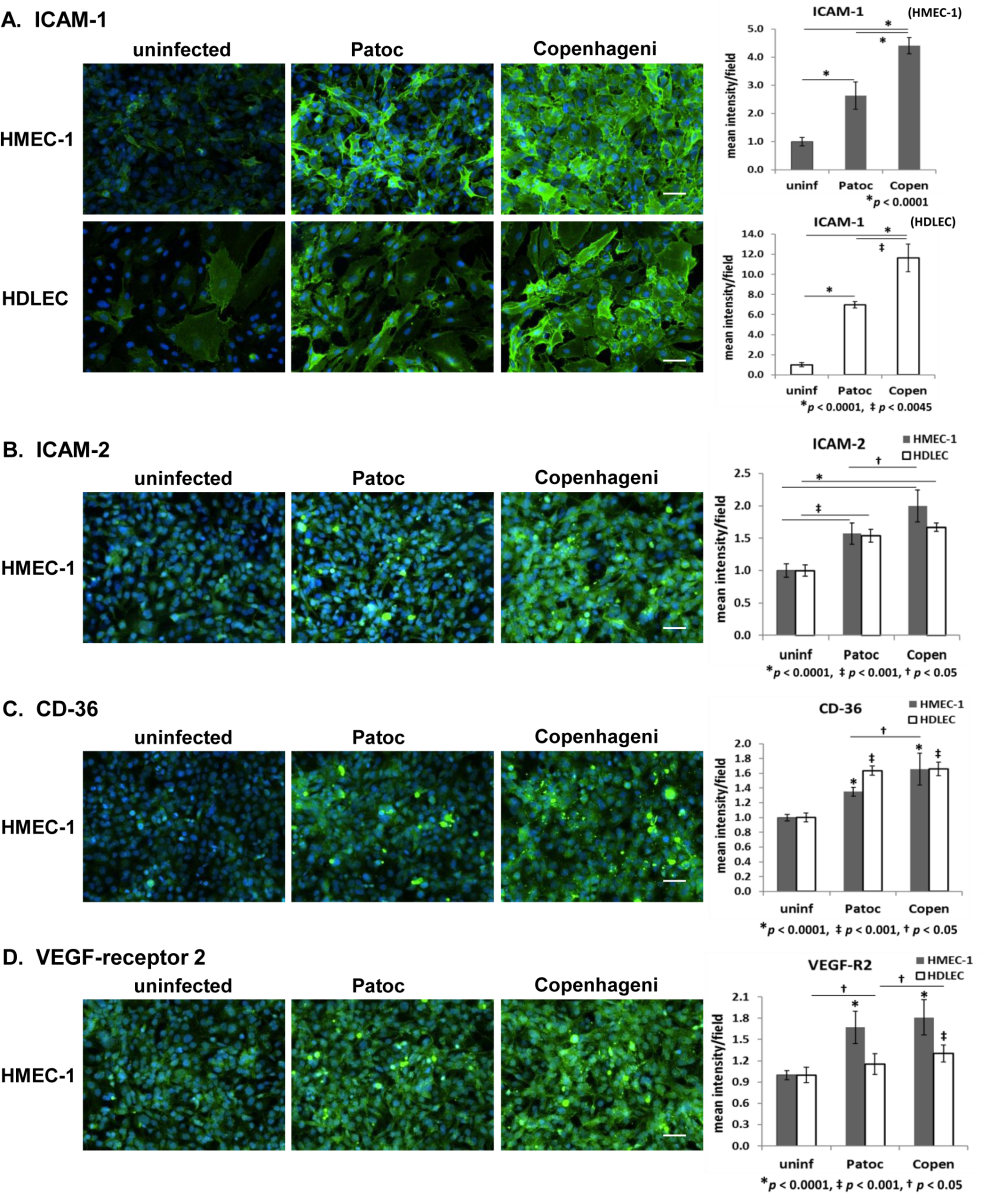
Effect of *Leptospira* infection on ICAMs and cell surface receptors in endothelial cells. (A) ICAM-1 (4-fold longer exposure time was used for uninfected HDLEC), (B) ICAM-2, (C) CD-36, and (D) vascular endothelial growth factor-receptor 2 (VEGF-R2) in HMEC-1 and HDLEC are shown in green. The nuclei are stained in blue for all panels. Scale bars represent 50 μm. Quantified signal intensity of the host protein is indicated in the right-hand graphs (mean +/- SD, *p*-value is indicated below each graph, the independent *p*-values shown as an asterisk or a double-dagger are compared to uninfected cells).

In addition to ECM proteins, other host cell surface proteins may be involved in *Leptospira* infection or pathogenicity. To test this hypothesis, we examined intercellular adhesion molecules (ICAMs) and other cell surface receptors. ICAMs belong to the immunoglobulin superfamily, and participate in inflammatory responses [49]. Compared to uninfected endothelial cells, the signal intensities of ICAM-1 and ICAM-2 were elevated by infection with either *L*. *interrogans* sv. Copenhageni or *L*. *biflexa* sv. Patoc (Fig 2A and 2B, S2A Fig). The signal increase was more apparent with Copenhageni infection, especially for ICAM-1 in both cell types: a 4.5-fold increase in HMEC-1 and a 12-fold in HDLEC (Fig 2A, Table 2). For the ICAM-2 signal, Copenhageni infection caused an increase of 1.6- to 2.2-fold in endothelial cells (Fig 2B, S2A Fig). Patoc infection demonstrated an intermediate increase in both ICAM-1 and ICAM-2 (Fig 2A and 2B, S2A Fig).

CD36 is a fatty acid/scavenger receptor, and is involved in microvascular endothelial cell migration and metastasis [50, 51]. *Leptospira* infection increased the signal intensity of CD36 by 1.3- to 1.8-fold, and was slightly higher in Copenhageni-infected than Patoc-infected HMEC-1 (Fig 2C). Infection also elevated the CD36 signal in HDLEC, although the difference between Copenhageni- and Patoc was miniscule, and mainly caused by the increase in punctate morphology (Fig 2C, S2B Fig). These data suggest that this CD36 phenotype is induced by *Leptospira* infection with both pathogenic and non-pathogenic strains.

Vascular endothelial growth factor-receptor 2 (VEGF-R2) is another *Leptospira*-influenced cell surface protein. This VEGF-specific receptor is involved in the proliferation of vascular endothelial cells and the regulation of the endothelial barrier function [52]. *Leptospira* infection with both Copenhageni and Patoc elevated the VEGF-R2 signal, more so in HMEC-1 (1.5- to 2-fold) than the slight increase (1.1- to 1.4-fold) in HDLEC (Fig 2D, S2C Fig). Again, these signal elevations in VEGF-R2 were induced by both pathogenic and non-pathogenic *Leptospira* species.

### Intracellular proteins–VEGF, RhoA, and ILK (integrin-linked kinase)

**Fig 3:**
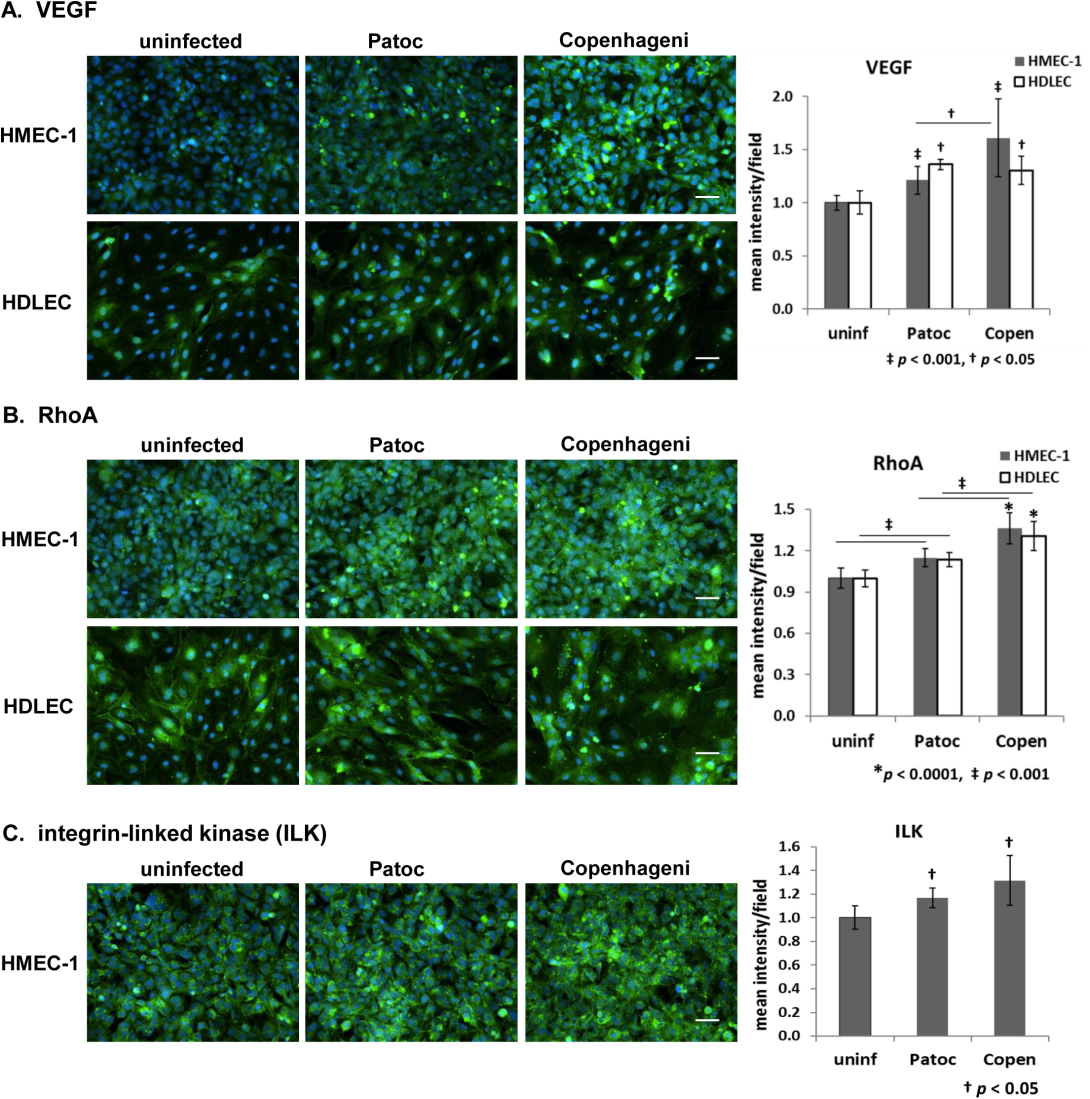
Effect of *Leptospira* infection on intracellular proteins in endothelial cells detected by immunofluorescence microscopy. (A) vascular endothelial growth factor (VEGF), (B) small GTPase RhoA, and (C) integrin-linked kinase (ILK) in HMEC-1 and HDLEC are shown in green. The nuclei are stained in blue for all panels. Scale bars represent 50 μm. Quantified signal intensity of the host protein is indicated in the right-hand graphs (mean +/- SD, *p*-value is indicated below each graph, the independent *p*-values shown as an asterisk, a dagger, or a double-dagger are compared to uninfected cells).

Although the screening of cell surface proteins was originally our focus, we also examined several intracellular proteins. We found three intracellular host proteins that were affected by *Leptospira* infection (Table 2). One protein was vascular endothelial growth factor (VEGF), which plays roles in the control of vascular endothelial cell proliferation and vascular permeability [53]. Infection with either *L*. *interrogans* sv. Copenhageni or *L*. *biflexa* sv. Patoc slightly elevated the fluorescence signal of VEGF by 1.1- to 1.9-fold with puncta formation (Fig 3A). The signal increase caused by the pathogenic Copenhageni was higher than by the nonpathogenic Patoc in HMEC-1 but there was no difference between the changes caused by the two *Leptospira* strains in HDLEC (Fig 3A).

A second *Leptospira*-affected protein, the small GTPase RhoA, is an important molecule that regulates the assembly of the actin cytoskeleton and the remodeling of cell junction proteins. RhoA activity accompanied by actin remodeling can lead to a loss of endothelial barrier integrity [52, 54–56]. In both endothelial cell types we tested, the fluorescence intensity of small GTPase RhoA was slightly elevated by Copenhageni (1.3- to 1.4-fold) and Patoc (1.1- to 1.2-fold) infection (Fig 3B). The signal increase was slightly higher when cells were infected with Copenhageni than with Patoc (Fig 3B). An increase in punctate morphology of the RhoA signal was also observed in infected cells, but not specific to the *Leptospira* species (Fig 3B).

Another intracellular protein affected by *Leptospira* infection is integrin-linked kinase (ILK). ILK associates with integrins as a regulator of integrin-mediated signaling, correlating with multiple cellular functions such as cell proliferation, migration, adhesion, and vascular integrity [57–59]. The intensity of ILK signal was slightly higher (1.1- to 1.5-fold) with puncta formation when HMEC-1 were infected with leptospires regardless of pathogenic status (Fig 3C).

Overall, *Leptospira*-mediated changes were found in intracellular host proteins that have roles in the regulation of cell proliferation, endothelial barrier integrity, and actin remodeling, but differences between the pathogenic and non-pathogenic strains were not always apparent.

### Cell-cell junction proteins

In endothelial cells, intercellular connections are formed through multiple adhesive structures, regulating the passage of blood constituents and circulating cells to the underlying tissues [60, 61]. Pathological conditions of endothelial paracellular permeability lead to severe or fatal organ dysfunction [60, 61], similar to the symptoms in severe leptospirosis patients. To determine the effect of *Leptospira* infection on cell-cell junctions, we examined the transmembrane proteins and cytosolic adaptor proteins of three major intercellular junction types: 1) adherens junction, 2) tight junction, and 3) gap junction.

#### Adherens junction–VE-cadherin, p120 catenin, alpha-catenin, and beta-catenin

Adherens junctions (or zonula adherens, intermediate junctions) are composed of adhesion protein complexes located at the basal side of cell–cell junctions, playing a significant role in endothelial barrier function [60–63]. VE-cadherin (vascular endothelial cadherin, also known as cadherin 5 or CD144) is exclusively expressed in endothelial cells, and is essential for the formation of adherens junctions and the endothelial barrier [60, 63]. This transmembrane protein forms homodimers on the cell surface, interconnecting neighboring endothelial cells [62, 64, 65]. The cytoplasmic face of VE-cadherin associates directly with p120 catenin and beta-catenin, and indirectly with alpha-catenin and the actin cytoskeleton, forming the adherens junction as a stable intercellular- and intracellular-structure [62, 66].

**Fig 4:**
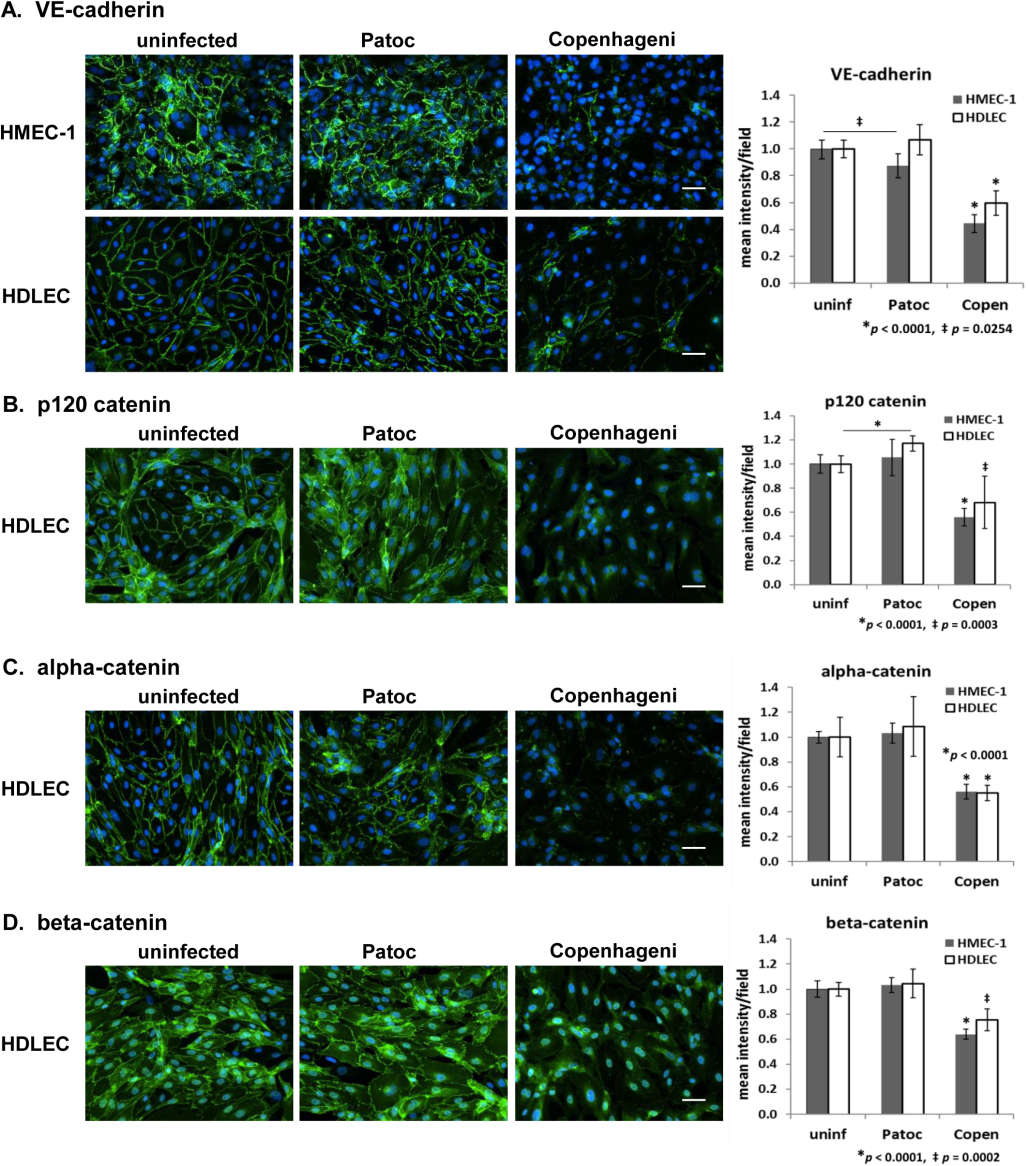
Effect of *Leptospira* infection on adherens junction proteins in endothelial cells detected by immunofluorescence microscopy. (A) VE-cadherin, (B) p120 catenin, (C) alpha-catenin, and (D) beta-catenin in HMEC-1 and HDLEC are shown in green. The nuclei are stained in blue for all panels. Scale bars represent 50 μm. Quantified signal intensity of the host protein is indicated in the right-hand graphs (mean +/- SD, *p*-value is indicated below or inside the graph, the independent *p*-values shown as an asterisk or a double-dagger for Copenhageni are compared to uninfected and Patoc-infected cells).

In our experiments, VE-cadherin was clearly localized at intercellular junctions of uninfected cells, especially in HDLEC (Fig 4A, uninfected). When endothelial cells were infected with *L*. *interrogans* sv. Copenhageni, the fluorescence intensity of VE-cadherin was dramatically reduced by 40 to 60% as compared to that of uninfected cells, and was largely lost at the original localization at cell-cell junctions (Fig 4A, Table 2). In comparison, infection with *L*. *biflexa* sv. Patoc or pathogenic *B*. *burgdorferi* B31-A3 did not demonstrate either prominent signal reduction or mislocalization of VE-cadherin (Fig 4A, S9 Fig).

Infection with pathogenic *L*. *interrogans* sv. Copenhageni decreased VE-cadherin signal in endothelial cells at MOI of 10, 15, or 20 (S3A Fig). With another pathogen, *L*. *interrogans* sv. Canicola, the effect of infection on endothelial cells was weaker, with only MOIs of 15 and 20 demonstrating apparent reduction in VE-cadherin, and the level of reduction was dose-dependent (S3A Fig). There were no detectable changes at early time points, e.g. 7 h post inoculation (hpi) or earlier, when HMEC-1 were infected with Copenhageni at an MOI of 20 (S3B Fig). The saprophyte *L*. *biflexa* sv. Patoc demonstrated little influence on VE-cadherin signal at MOIs of 10, 15, and 20 (S3A Fig). Although it is known that VE-cadherin can be internalized via endocytosis as a part of regulation of endothelial barrier function [52, 53], we did not observe the internalization of VE-cadherin even when cells were permeabilized to allow examination of possible intracellular localization of this protein (S3C Fig).

Intracellular adapter proteins that associate with VE-cadherin at the adherens junction include p120 catenin, alpha-catenin, and beta-catenin [62, 67]. p120 catenin and beta-catenin directly interact with the cytoplasmic face of VE-cadherin while alpha-catenin indirectly associates as a component of the adherens junction complex [62, 66]. p120 catenin possesses a key role in VE-cadherin expression, internalization, and membrane localization as well as the regulation of endothelial permeability [63, 68]. Infection with the pathogenic Copenhageni reduced the fluorescence intensity of p120 catenin by 30 to 40% of the signal detected in uninfected endothelial cells (Fig 4B, S4A Fig, Table 2). As observed with VE-cadherin, Copenhageni infection disrupted the localization of p120 catenin at intercellular junctions (Fig 4B, S4A Fig). Infection with the non-pathogenic Patoc did not alter the junctional localization p120 catenin (Fig 4B, S4A Fig).

Alpha-catenin functions as a molecular switch through exclusively associating with either an cadherin/beta-catenin complex or actin filaments, coordinating actin organization and remodeling [66, 69]. The signal intensity of alpha-catenin was significantly decreased by Copenhageni infection even though alpha-catenin associates with VE-cadherin indirectly (Fig 4C, S4B Fig). The fluorescence signal was reduced to approximately 50% of the signal detected in uninfected endothelial cells, and alpha-catenin was almost invisible at the cell periphery (Fig 4C, S4B Fig). As observed in VE-cadherin and p120 catenin, there was little influence by infection with the saprophyte Patoc strain (Fig 4C, S4B Fig).

Beta-catenin is a multifunctional protein that localizes at either the cadherin adhesive complex in cell-cell junctions or at the T-cell factor (TCF)-transcriptional complex in the nucleus of multiple types of cells, including endothelial cells [65, 70]. VE-cadherin/beta-catenin complexes are involved in the regulation of endothelial cell survival as well as vascular patterning and permeability [71, 72]. In uninfected cells, beta-catenin was localized at the cell-cell junction and in the nucleus as expected (Fig 4D, see single-color format in S5 Fig). In cells infected with Copenhageni, beta-catenin was reduced at the cell-cell junction, although the localization in the nucleus was unchanged and remained at the levels in uninfected or Patoc-infected cells (Fig 4D, S4C Fig and S5 Fig). Overall, the fluorescence signal of beta-catenin decreased by 20–30% as compared to uninfected cells (Fig 4D, S4C Fig). The signal reduction was not as intense as VE-cadherin, p120 catenin, or alpha-catenin, which is likely the result of the unchanged fluorescence signal in the nucleus regardless of *Leptospira* infection.

Nectin is another type of an adherens junction protein that functions independently from VE-cadherin complexes, and can be located near tight junctions during junctional development [64, 65]. While VE-cadherin and its adaptor protein catenins at the adherens junction were largely decreased by Copenhageni infection (Fig 4, S4 Fig), the overall morphology of nectin was not affected (S6 Fig). In contrast to other adherens junction proteins, the signal intensity of nectin subtly increased in cells infected with either Copenhageni or Patoc (S6 Fig). These data suggest that pathogenic *Leptospira* specifically targets the adherens junctions containing the VE-cadherin/catenins complex.

#### Tight junction–zonula occludens-1 (ZO-1)

Another group of adhesive junctional structures is tight junctions (occluding junctions or zonula occludens), which are generally located at the apical side of cell-cell junctions as compared to adherens junctions [62, 64]. Major types of tight junction proteins include claudin, occludin, and zonula occludens-1 (ZO-1) [64, 73]. Claudin and occludin are transmembrane proteins, directly involved in junctional adhesion [62, 64]. We examined the effect of *Leptospira* infection on claudin 5 and occludin. Claudin 5 is specifically produced in endothelial cells and the expression is partially controlled by VE-cadherin complexes [65]. During infection with either *L*. *interrogans* sv. Copenhageni and *L*. *biflexa* sv. Patoc, there was little or no change in morphology or the signal intensity of claudin 5 or occludin (S7 Fig).

**Fig 5:**
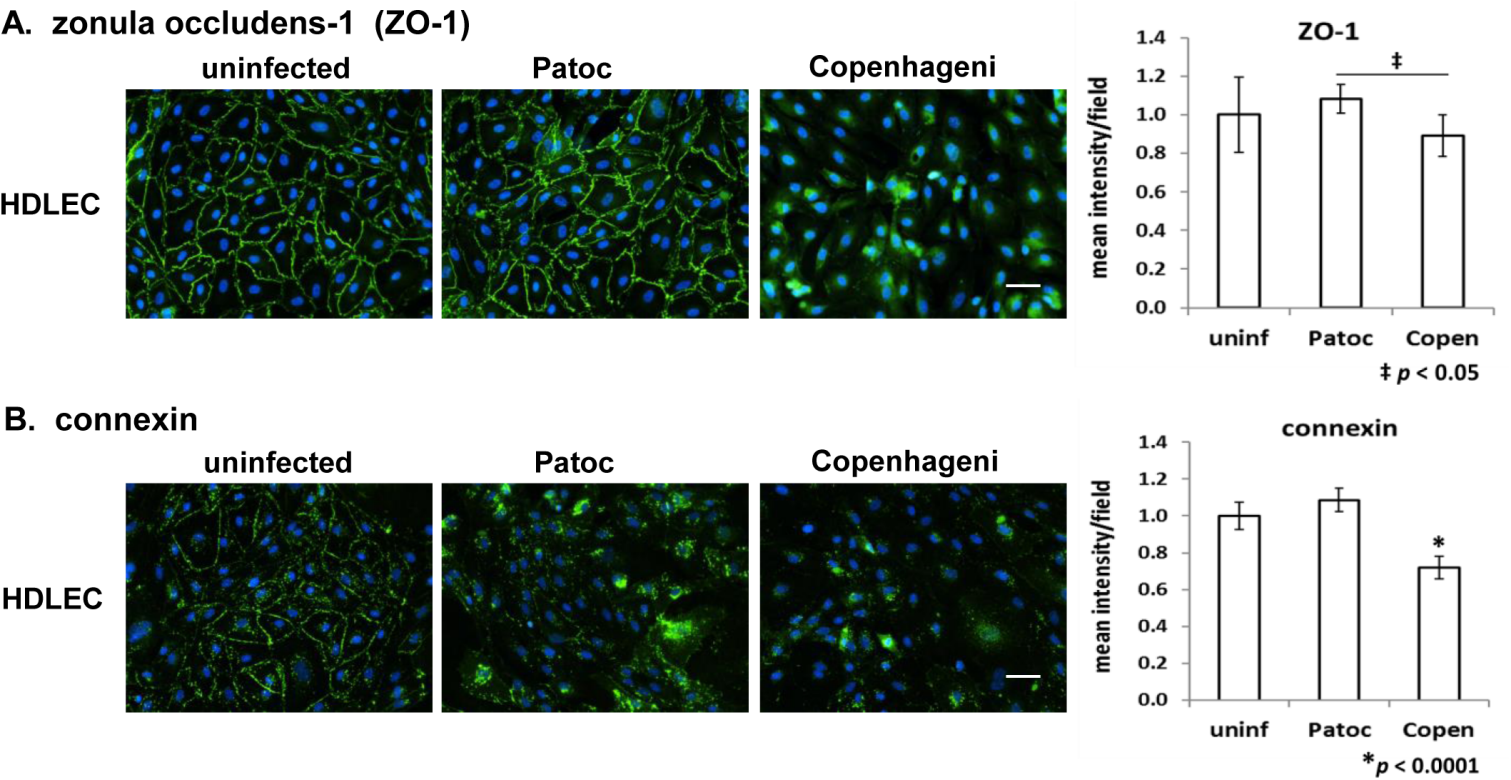
Effect of *Leptospira* infection on tight junction and gap junction proteins in endothelial cells. (A) tight junction protein, zonula occludens-1 (ZO-1) and (B) gap junction protein, connexin 43 (connexin) in HDLEC are shown in green. The nuclei are stained in blue for all panels. Scale bars represent 50 μm. Quantified signal intensity of the host protein is indicated in a right-hand graph (mean +/- SD, *p*-value is indicated below each graph, the independent *p*-value shown as an asterisk for Copenhageni is compared to uninfected and Patoc-infected cells).

ZO-1 is a peripheral membrane protein located at the cytoplasmic side of the plasma membrane [64, 73]. In uninfected HDLEC, ZO-1 was apparently localized at cell-cell junctions (Fig 5A, uninfected). In contrast to the results with claudin 5 and occludin (S7 Fig), Copenhageni infection clearly disrupted the localization of ZO-1 from the cell periphery (Fig 5A, Table 2). The fluorescence signal of ZO-1 indicated relocalization of this protein from cell-cell junctions to intracellular locations, as the total signal intensity decreased only slightly (Fig 5A). Patoc infection did not influence the intercellular junction localization or signal intensity of ZO-1 (Fig 5A). In summary, the intracellular tight junction protein ZO-1 was mislocalized by the pathogenic *Leptospira* species but the transmembrane proteins claudin 5 and occludin, which play an essential role in junctional adhesion, were not affected.

#### Gap junction–Connexin

A gap junction is formed as junctional channels between neighboring cells by oligomers of specific integral membrane proteins, such as connexins [74, 75]. The gap junctional proteins play roles in subcellular microdomain signaling as well as the regulation of intercellular communication through passage of ions and small molecules [74, 75]. The gap junction protein we examined is connexin 43, which is the most ubiquitously distributed of this class of proteins in mammalian cells [74].

To investigate the gap-junction structure in endothelial cells, HDLEC cells, which possess well-organized cell junctions, were used. In uninfected cells, connexin 43 localized at intercellular junctions in a punctate signal pattern, suggesting a less well-organized cell junction morphology (Fig 5B, uninfected), as compared to adherens junctions (Fig 4) and tight junctions (ZO-1, Fig 5A). *L*. *interrogans* sv. Copenhageni infection decreased the signal intensity of connexin 43 more than 20% as compared to uninfected cells, especially at the cell periphery (Fig 5B, Table 2). *L*. *biflexa* sv. Patoc infection demonstrated translocation of connexin 43 from the cell-cell junctions to intracellular locations, but the overall signal intensity was not significantly influenced (Fig 5B).

### Cytoskeletal protein–Filamentous actin

**Fig 6:**
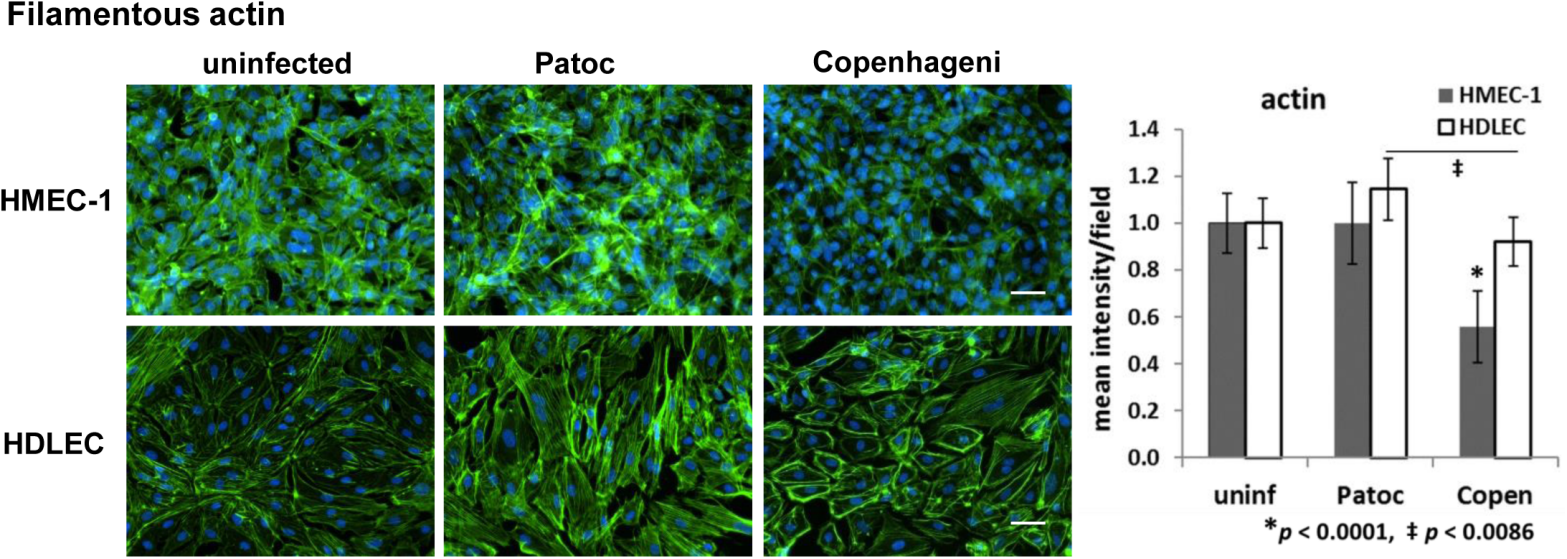
Effect of *Leptospira* infection on actin filaments in endothelial cells detected by immunofluorescence microscopy. Actin filaments in HMEC-1 and HDLEC are shown in green. The nuclei are stained in blue for all panels. Scale bars represent 50 μm. Quantified signal intensity of the host protein is indicated in the right-hand graph (mean +/- SD, *p*-value is indicated below the graph, the independent *p*-value shown as an asterisk for Copenhageni is compared to uninfected and Patoc-infected cells).

*L*. *interrogans* sv. Copenhageni infection apparently disrupted multiple proteins of the adherens junction, the tight junction protein ZO-1, and the gap junction protein connexin (Figs 4 and 5). The adherens junction and tight junction proteins directly or indirectly interact with actin filaments to stabilize the cellular structure and cell junctions [72, 73]. To examine if *Leptospira* infection influences actin filaments (microfilaments) in endothelial cells, filamentous actin was labeled with Alexa Fluor-conjugated phalloidin. In permeabilized, uninfected HMEC-1, actin filaments were visualized as net-shaped structures, spreading ubiquitously in the cell (Fig 6, uninfected). When this cell type was infected with Copenhageni, the signal intensity of actin filaments decreased to ~50% of uninfected cells (Fig 6, Table 2). *L*. *biflexa* sv. Patoc infection demonstrated no reduction in signal intensity (Fig 6).

In another type of endothelial cells, HDLEC, the morphology of actin filaments appeared well-organized, with long straight filaments rather than the net-shaped morphology (Fig 6A, uninfected). In contrast to the reduction of the actin signal in Copenhageni-infected HMEC-1, the signal decrease in HDLEC was minor (Fig 6). Instead, Copenhageni infection induced a morphological rearrangement of actin filaments: intense localization of filamentous actin at the cell periphery and reduction of stress fibers inside the cell (Fig 6, Table 2). Patoc infection induced slightly more stress fibers but did not affect the overall morphology and signal intensity of filamentous actin in HDLEC (Fig 6). Throughout our screening of host proteins, only this protein demonstrated host cell-type specific changes in morphology and phenotype.

We also examined another cytoskeletal structure, the microtubule, by immune-labeling the most critical protein, alpha-tubulin (Table 1). There was no detectable change in morphology or signal-intensity in HMEC-1 or HDLEC infected with either Copenhageni or Patoc (S8 Fig). These data suggest that the pathogenic *Leptospira* specifically modifies the actin filaments of the cytoskeleton.

## Discussion

To investigate the pathogenic effects of *Leptospira* infection of human endothelial cells, we utilized immunofluorescence microscopy to screen for changes in host protein abundance and distribution. Seventeen proteins indicated minor to major changes in *Leptospira*-infected endothelial cells. These 17 proteins are classified into five biological groups: 1) extracellular matrix, 2) intercellular adhesion molecules and cell surface receptors, 3) intracellular proteins, 4) cell-cell junction proteins, and 5) a cytoskeletal protein.

The most prominent phenotype of infection with pathogenic *L*. *interrogans* sv. Copenhageni was the dramatically reduced multiple adherens junction proteins and one of the tight junction proteins (ZO-1), a gap junction protein (connexin) as well as filamentous actin (summarized in Table 2). Infection with *Leptospira*, regardless of the strain’s pathogenicity or ability to harm host cells, increased the signal intensity of some of the ECM proteins, ICAMs, cell surface receptors, and intercellular proteins (Table 2). In general, the signal increase was more intense when endothelial cells were infected with pathogenic *L*. *interrogans* sv. Copenhageni than the saprophyte strain, *L*. *biflexa* sv. Patoc (Table 2).

Among ECM proteins, collagen type IV, decorin, and laminin were influenced by *Leptospira* infection (Fig 1). It has been reported that outer membrane protein(s) of pathogenic *Leptospira* can increase the production of an ECM protein, collagen type IV [76]. In this study, the signal elevations of collagen and decorin were caused by both pathogenic and non-pathogenic leptospires (Fig 1A and 1B). Kassegne et al. reported that *L*. *interrogans* possesses a collagenase, which is involved in the invasion and transmission of the pathogenic species [77]. Morphological changes we observed, formation of puncta as a result of infection (Fig 1A), may be a result of protein degradation by collagenase activity, which could expose epitopes for antibody binding, increasing the fluorescence signal. For laminin, the structural rearrangement was specifically caused by the pathogenic Copenhageni (Fig 1C). The net-shape structure of laminin appeared rolled up, forming thick bundles with little change in total signal intensity, suggesting that this phenotype could be a secondary effect of the Copenhageni-mediated disassembly of adherens junctions.

*Leptospira* infection influenced several cell-surface proteins/receptors, ICAM-1, ICAM-2, CD36, and VEGF-receptor 2 (Fig 2, S2 Fig, Table 2). We observed a significant signal increase of ICAMs in both HMEC-1 and HDLEC when infected with leptospires and the increase was more prominent with Copenhageni in HMEC-1 (Fig 2). Because ICAMs cluster at intercellular junctions distinct from the adherens junction and tight junction [61], the elevation of ICAMs is likely an independent phenomenon from the disruption of adherens junctions by pathogenic *Leptospira*. In pulmonary leptospirosis patients, an increase in ICAM-1 expression was detected in the alveolar septa and pulmonary vessels [7]. However, it was also reported that there was no significant change in ICAM-1 cell surface expression in HUVEC after 24 h and 48 h infection as detected by horseradish peroxidase reaction [78]. This difference may be due to the sources of the cells or specific experimental conditions, as other work has shown that leptospiral lipopolysaccharide (LPS) and outer membrane proteins increase ICAM-1 expression in HUVECs [38, 39]. During early stages of leptospirosis, leptospiral LPS and outer membrane lipoproteins induce inflammation primarily via Toll-like receptor 2 (TLR2) and in some experiments, via TLR4 activation [33, 34, 79–81]. Interestingly, pathogenic *Leptospira* infection induces pro-inflammatory reactions in human (susceptible to leptospirosis) but activates anti-inflammatory pathways in mice (not susceptible to clinical leptospirosis) [18, 36]. Thus, the phenotype of ICAM increase in human endothelial cells may be a result of inflammatory reaction induced by TLR-mediated signaling pathways and other pro-inflammatory responses.

The fatty acid/scavenger receptor CD36 is involved in the regulation of microvascular endothelial cell migration and is implicated as having a role in inflammation [50, 51, 82]. CD36 is also known to interact with collagens [83]. Since we observed *Leptospira*-mediated changes in collagen type IV and type VI (Fig 1A and S1A Fig), the signal increase phenotype of CD36 that we observed could be induced by multiple factors, such as changes in collagen or inflammatory signaling. Another receptor, VEGF-R2, specifically reacts to VEGF in controlling the growth of vascular endothelial cells. Another function of VEGF-R2 is that this receptor interacts with VE-cadherin, which physically limits the internalization of VE-cadherin from adherens junctions [52]. Overproduction of VEGF-R2 might inhibit the internalization of VE-cadherin, but we observed a reduction in VE-cadherin signal without apparent internalization (Fig 4A and S3C Fig). The elevation of the VEGF-R2 signal may be a response to leptospire-mediated VEGF increase rather than a direct response to *Leptospira* contact with endothelial cells.

*Leptospira* infection increased the fluorescence signal and puncta formation of three intracellular proteins, VEGF, RhoA, and ILK (Fig 3, Table 2). In addition to its function in vascular endothelial growth, VEGF promotes vascular permeability *via* the phosphorylation and endocytosis of VE-cadherin; this modulation is reversible [61]. There was no detectable internalization of VE-cadherin (S3C Fig) and the signal increase of VEGF was not specific to the pathogenic strain (Fig 3A), implying that any involvement of VEGF in pathogenicity is less likely. ILK is a regulator of integrin-mediated signaling to regulate cell migration, adhesions, and vascular integrity [57–59]. The *Leptospira*-mediated changes were statistically significant but modest and also detected in cells infected both Copenhageni and Patoc strains (Fig 3C).

Endothelial permeability is controlled by the opening and closing of cell-cell junctions via the rearrangement of junction proteins and cytoskeletal proteins [61]. To regulate the adherens junction organization and endothelial permeability, some small GTPases are involved [53, 84]. For example, the small GTPase RhoA controls the endothelial barrier integrity via remodeling of the actin cytoskeleton and of junction proteins [52, 54–56]. In this study, *Leptospira* infection altered the actin cytoskeleton along with a moderate elevation of the RhoA signal (Figs 6 and 3B). The increase of RhoA signal was not specific to Copenhageni infection, though the signal intensity was higher than with Patoc inoculation (Fig 3B). These data suggest that the involvement of these intracellular proteins in the *Leptospira* pathogenicity, especially in disrupting the endothelial integrity, is relatively minor.

**Fig 7:**
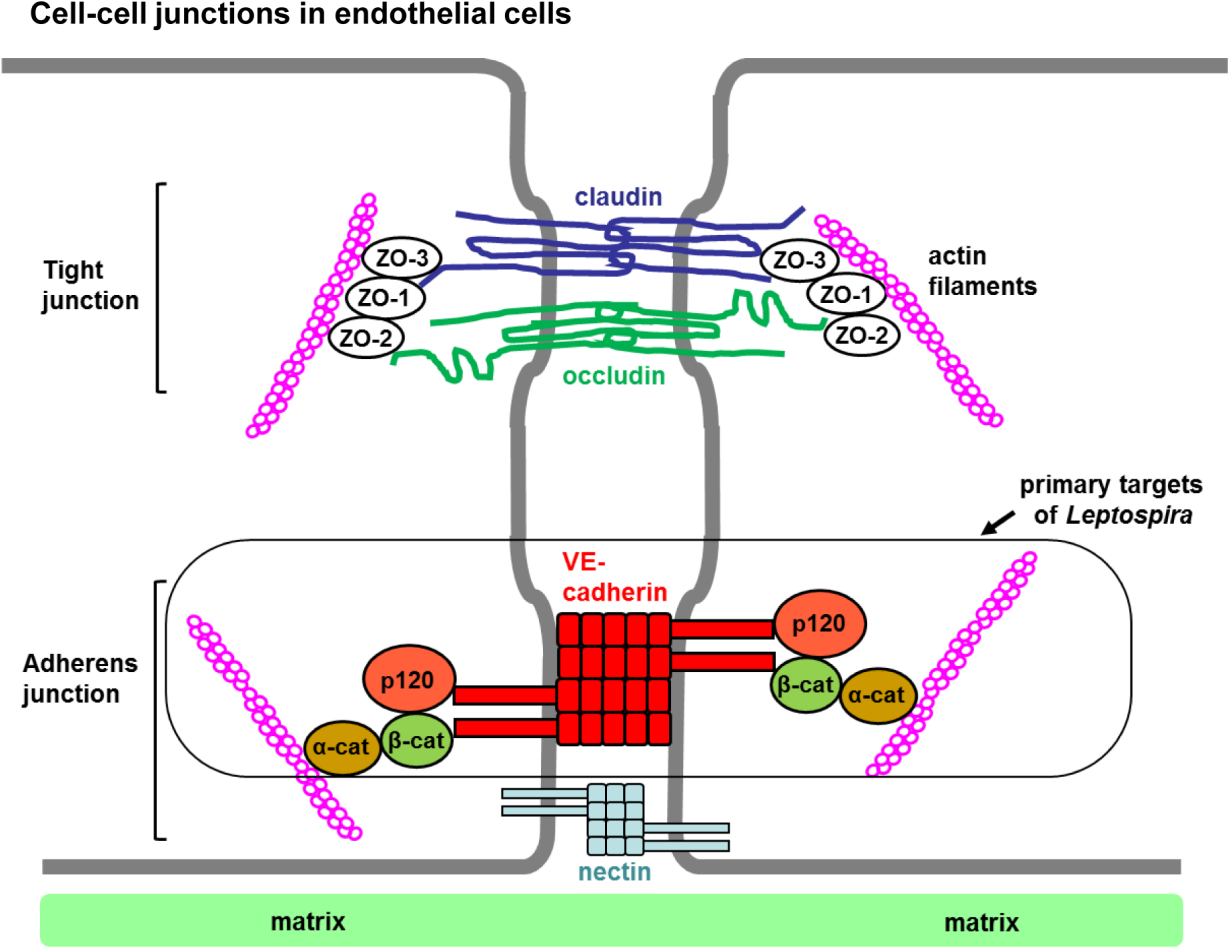
Features of intercellular junctions in endothelial cells. The tight junction consists of claudin, occludin, and intracellular zonula occludens (ZO) proteins. ZO-1, ZO-2, and ZO-3 directly associate with actin filaments. Pathogenic *Leptospira* infection disrupted only the ZO-1 structure in tight junctions. The adherens junction is comprised of VE-cadherin, p120 catenin (p120), alpha-catenin (α-cat), beta-catenin (β-cat), and nectin. Alpha-catenin is a molecular switch that interacts with either the cadherin/beta-catenin complex or actin filaments. Our data indicate that the primary target of pathogenic *Leptospira* is the VE-cadherin/catenins complex in adherens junctions.

In this study, the most prominent *L*. *interrogans* pathogenic phenotype was the disruption of adherens junctions (Fig 4, S4 Fig). The adherens junction proteins, VE-cadherin, p120 catenin, alpha-catenin, and beta-catenin, showed drastically reduced immunofluorescence signals specifically at the cell-cell junctions (Fig 4, S4 Fig; also see Fig 7 for structural features). Because Copenhageni-infection did not disturb occludin and claudin (tight junction markers, S7 Fig), the VE-cadherin/catenin-complex at the adherens junction is likely to be the main target of pathogenic *Leptospira* species in endothelial cells (Fig 7).

VE-cadherin is essential for adherens junction formation and barrier maintenance in endothelial cells, playing a critical role in vascular morphogenesis, especially remodeling and maturation [71, 85]. The administration of the anti-VE-cadherin antibody BV13 redistributes VE-cadherin molecules at adherens junctions in cultured endothelial cells and increases vascular permeability in heart and lungs of mice [60]. VE-cadherin-associated catenins are also important for the formation of the dynamic endothelial barriers. For example, VE-cadherin/beta-catenin complexes are involved in the regulation of endothelial cell survival, and in VE-cadherin mutant cells, beta-catenin was not localized at intercellular junctions [71]. Inactivation of the beta-catenin gene disrupted the cell junctional organization by reduction in alpha-catenin expression and cell-adhesion strength, leading to hemorrhagic vessels [72]. Also, p120 catenin is involved in maintaining VE-cadherin expression [68]. Thus, VE-cadherin and catenins are interconnected physically and functionally to regulate the expression and interaction of these proteins, maintaining the stability and flexibility of endothelial junctional barriers required for normal biological function. Our data indicate that pathogenic *Leptospira* strains target these adherens junction proteins important for the endothelial barrier integrity and disassemble the junction-protein complex (Fig 7).

In contrast to the drastic disturbance of the adherens junction (Fig 4, S4 Fig), there were no detectable changes in the tight junctional transmembrane proteins claudin 5 and occludin in endothelial cells infected with *L*. *interrogans* sv. Copenhageni (S7 Fig). These tight junction markers are less critical to the vascular barrier integrity since the deletion of the genes encoding claudin 5 or occludin does not influence on the vascular morphology and barrier function in mice [86, 87]. In our experiments, only ZO-1, a cytosolic peripheral protein, was apparently mislocalized from tight junctions (Fig 5A). Reduction of ZO-1 was previously observed in *Leptospira*-infected HUVEC [24]. ZO-1 is a tight junction protein when intercellular junctions are mature but localizes at adherens junctions at an early stage of cell-cell contact [62, 73, 88]. In the immature cell junctions, ZO-1 can directly associate with alpha-catenin and the actin cytoskeleton [67, 89] and indirectly associates with beta-catenin [90]. In the mature tight junction, ZO-1 indirectly influences the endothelial integrity *via* association with claudin, occuldin, and actin filaments [60, 75]. The published information and our data imply that *Leptospira* primarily disrupts the adherens junctions, resulting in mislocalization of one of the actin-binding proteins, ZO-1 (Fig 7).

A gap junction protein, connexin 43, was also mislocalized and showed reduced signal at cell junctions in endothelial cells when infected with pathogenic Copenhageni (Fig 5B). The non-pathogenic Patoc strain caused translocation of connexin 43 from the cell periphery to intracellular locations without losing signal intensity (Fig 5B). Connexin 43 associates with a variety of proteins located at adherens junctions and tight junctions, the cytoskeleton, and actin-binding proteins, including p120 catenin, beta-catenin, and ZO-1 [74, 75]. Moreover, the gap junction is not directly involved in endothelial permeability [62]. These data imply that the gap junction is not a primary target of pathogenic *Leptospira* and that the phenotype we observed is likely induced as a secondary effect following the disruption of the adherens junction.

Infection with the pathogenic Copenhageni demonstrated cell-type-specific phenotypes in the actin cytoskeleton: 1) reduction of the actin signal in HMEC-1 and 2) bundling and rearrangement of filamentous actin structure in HDLEC (Fig 6). Actin filaments physically interact with multiple cell-junction proteins, which regulates the dynamic rearrangement of the actin-filament structure [62]. For instance, alpha-catenin interacts with either cadherin/beta-catenin complex or actin filaments, regulating actin assembly and organization [67, 69]. The inactivation of the beta-catenin gene influences the morphology of actin filaments in endothelial cells [72], and ZO-1 regulates the cortical cytoskeleton at cell junctions [73]. The phenotypes of Copenhageni infection, a decrease in filamentous actin in HMEC-1 and translocation of the bundled-actin filaments to the cell periphery of HDLEC (Fig 6), may be induced by Copenhageni-mediated disruption of the VE-cadherin-catenin complex.

One of the functions of filamentous actin is stabilizing or reorganizing the intercellular junctions through interacting with cell-junctional proteins [62]. The cadherin-catenin complex is known to dynamically influence the actin cytoskeleton and *vice versa*: filamentous actin is necessary for the regulation of endothelial opening/closing in addition to the stabilization of cell-junctions [61, 62]. We considered the possibility that filamentous actin is the primary target of pathogenic *Leptospira* infection, but the inhibition of typical actin distribution or mobilization at cell-cell junctions by cytochalasin D or jasplakinolide do not influence the dynamics of cadherin and alpha-catenin [67], suggesting that filamentous actin is unlikely to be the primary target of pathogenic *Leptospira*.

In physiologic conditions *in vivo*, dynamic and transient remodeling of intercellular junctions is well-controlled and critical to cellular maintenance, especially in endothelial cells [62, 91]. However, drastic and irreversible changes in endothelial junctions contribute to pathological endothelial permeability and leakage as well as vascular network disruption [62]. Miyahara *et al*. identified intact cell attachment with some disturbance of intercellular junctions in hepatocytes of pre-icteric hamsters, with cell detachment plus disrupted junctional association in icteric hamsters [23]. In leptospirosis patients, proinflammatory response and vascular damage are pathologic features of leptospirosis-associated pulmonary hemorrhage syndrome or acute lung injury [11, 12, 25]. Thus, in the later stage of severe leptospirosis, *Leptospira* infection and detrimental inflammatory responses, independent of TLR activation [33], overwhelm the cellular maintenance system, leading to devastating damage to cell-cell junctions and vascular systems of the host.

We used cultured human endothelial cells to investigate how *Leptospira* may lead to endothelial permeability and, by inference, vascular damage seen in human patients and susceptible animals. Our study demonstrated that the primary targets of *L*. *interrogans* are intercellular junctions, primarily adherens junctions. Other host proteins affected by *L*. *interrogans* infection may be indirectly impacted by the damage to a modification of the primary targets. The changes in host proteins that were impacted by non-pathogenic Patoc, though not as robust as those impacted by Copenhageni, may be a consequence of pro-inflammatory responses induced by *Leptospira* LPS, cell-surface proteins, or secreted proteins.

Our systematic analyses of host proteins in *Leptospira* infected-human endothelial cells demonstrated pathogen-specific phenotypes in the adherens junction, filamentous actin and actin-associated proteins. Several phenotypes were observed with infection with either the pathogen or the non-pathogen in multiple biological groups. These data suggest that this zoonotic agent may damage endothelial cells *via* multiple cascades or pathways, potentially leading to the increased vascular permeability followed by severe illness *in vivo*. In addition, morphological and quantitative analyses of infected human cells by immunofluorescence microscopy constitute a reliable method to investigate the pathogenicity and biological functions of *Leptospira* strains and specific proteins *in vitro*. Further work based on these results will contribute to our understanding of pathophysiological mechanisms of *Leptospira* infection.

## Supporting information

S1 Fig Effect of *Leptospira* infection on extracellular matrix proteins in endothelial cells detected by immunofluorescence microscopy. (A) collagen type VI and (B) fibronectin in HMEC-1 and HDLEC are shown in green. The nuclei are stained in blue for all panels. Scale bars represent 50 μm. Quantified signal intensity of the host protein is indicated in the right-hand graphs (mean +/- SD, *p*-value is indicated below each graph, the independent *p*-values shown as a dagger are compared to uninfected cells).
(TIF)

S2 Fig Effect of *Leptospira* infection on ICAM-2 and cell surface receptors in HDLEC detected by immunofluorescence microscopy. (A) ICAM-2, (B) CD-36, and (C) vascular endothelial growth factor-receptor 2 (VEGF-R2) in HDLEC are shown in green. The nuclei are stained in blue for all panels. Scale bars represent 50 μm. Quantified signal intensity of the host protein is indicated in the right-hand graphs (mean +/- SD, *p*-value is indicated below each graph, the independent *p*-values shown as an asterisk or a double-dagger are compared to uninfected cells).
(TIF)

S3 Fig Effect of *Leptospira* infection on VE-cadherin in endothelial cells in various experimental conditions. (A) infection of HMEC-1 at various MOIs, (B) early time point of infection (7 h post-inoculation), and (C) detection of VE-cadherin after methanol permeabilization. VE-cadherin in HMEC-1 and HDLEC is shown in green. The nuclei are stained in blue for all panels. Scale bars represent 50 μm.
(TIF)

S4 Fig Effect of *Leptospira* infection on adherens junction proteins in HMEC-1 detected by immunofluorescence microscopy. (A) p120 catenin, (B) alpha-catenin, and (C) beta-catenin in HMEC-1 are shown in green. The nuclei are stained in blue for all panels. Scale bars represent 50 μm. Quantified signal intensity of the host protein is indicated in the right-hand graphs (mean +/- SD, *p*-value is indicated below or inside the graph, the independent *p*-values shown as an asterisk or a double-dagger for Copenhageni are compared to uninfected and Patoc-infected cells).
(TIF)

S5 Fig Effect of *Leptospira* infection on the adherens junction protein beta-catenin in HDLEC. Beta-catenin is shown in green as a single color (top panels), nuclei are shown in blue as a single color (DAPI, middle panels), and overlay images (bottom panels). Scale bars represent 50 μm.
(TIF)

S6 Fig Effect of *Leptospira* infection on the adherens junction protein nectin in endothelial cells. Nectin 2 in HMEC-1 and HDLEC is shown in green. The nuclei are stained in blue for all panels. Scale bars represent 50 μm. Quantified signal intensity of the host protein is indicated in the right-hand graph (mean +/- SD, *p*-value is indicated below the graph).
(TIF)

S7 Fig Little effect of *Leptospira* infection on the tight junction proteins claudin and occludin in endothelial cells. (A) claudin 5 and (B) occludin in HDLEC are shown in green. The nuclei are stained in blue for all panels. Scale bars represent 50 μm. Quantified signal intensity of the host protein is indicated in the right-hand graphs. There was no significant difference in the signal intensity of claudin 5 or occludin between infected and uninfected cells.
(TIF)

S8 Fig Little effect of *Leptospira* infection on the microtubule protein alpha-tubulin in endothelial cells. Alpha-tubulin in HMEC-1 and HDLEC is shown in green. The nuclei are stained in blue for all panels. Scale bars represent 50 μm. Quantified signal intensity of the host protein is indicated in the right-hand graph. There was no significant difference in the signal intensity of alpha-tubulin between infected and uninfected cells.
(TIF)

S9 Fig No significant effect of *Borrelia* infection on VE-cadherin in endothelial cells. Endothelial cells were infected with the wild-type *Borrelia burgdorferi* B31-A3. VE-cadherin in HMEC-1 and HDLEC is shown in green. The nuclei are stained in blue for all panels. Scale bars represent 50 μm. Quantified signal intensity of the host protein is indicated in the right-hand graph. There was no significant difference in the signal intensity of VE-cadherin between infected and uninfected cells.
(TIF)
