# Supplementary figures and images for: Leptospira interrogans causes quantitative and morphological disturbances in adherens junctions and other biological groups of proteins in human endothelial cells
Source: PLoS Negl Trop Dis. 2017 Jul 27;11(7):e0005830. doi: 10.1371/journal.pntd.0005830 (PMC5549773; doi:10.1371/journal.pntd.0005830)

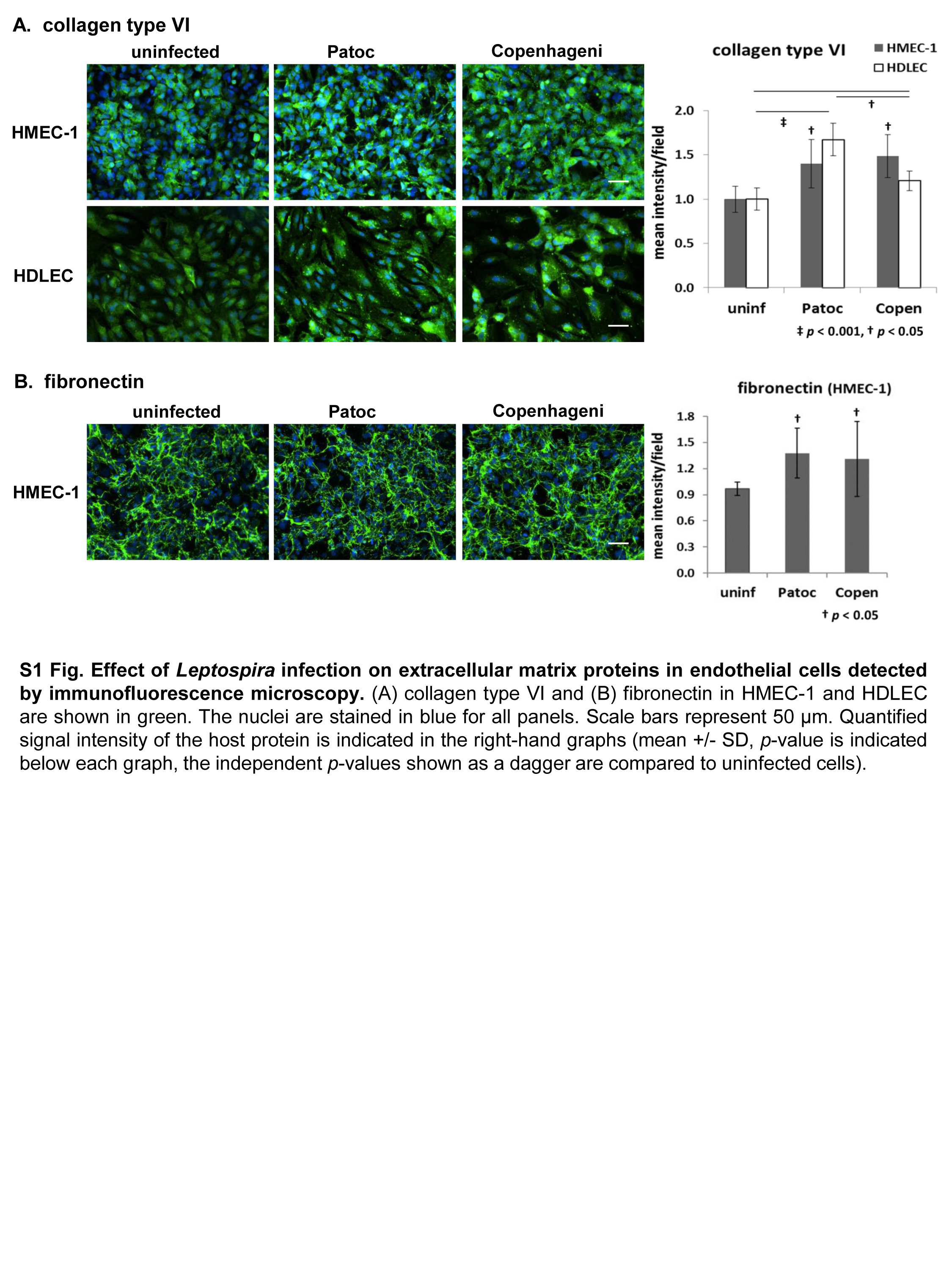

Supplement: S1 Fig — (A) collagen type VI and (B) fibronectin in HMEC-1 and HDLEC are shown in green. The nuclei are stained in blue for all panels. Scale bars represent 50 μm. Quantified signal intensity of the host protein is indicated in the right-hand graphs (mean +/- SD, p-value is indicated below each graph, the independent p-values shown as a dagger are compared to uninfected cells). (TIF) [file pntd.0005830.s001.tif]

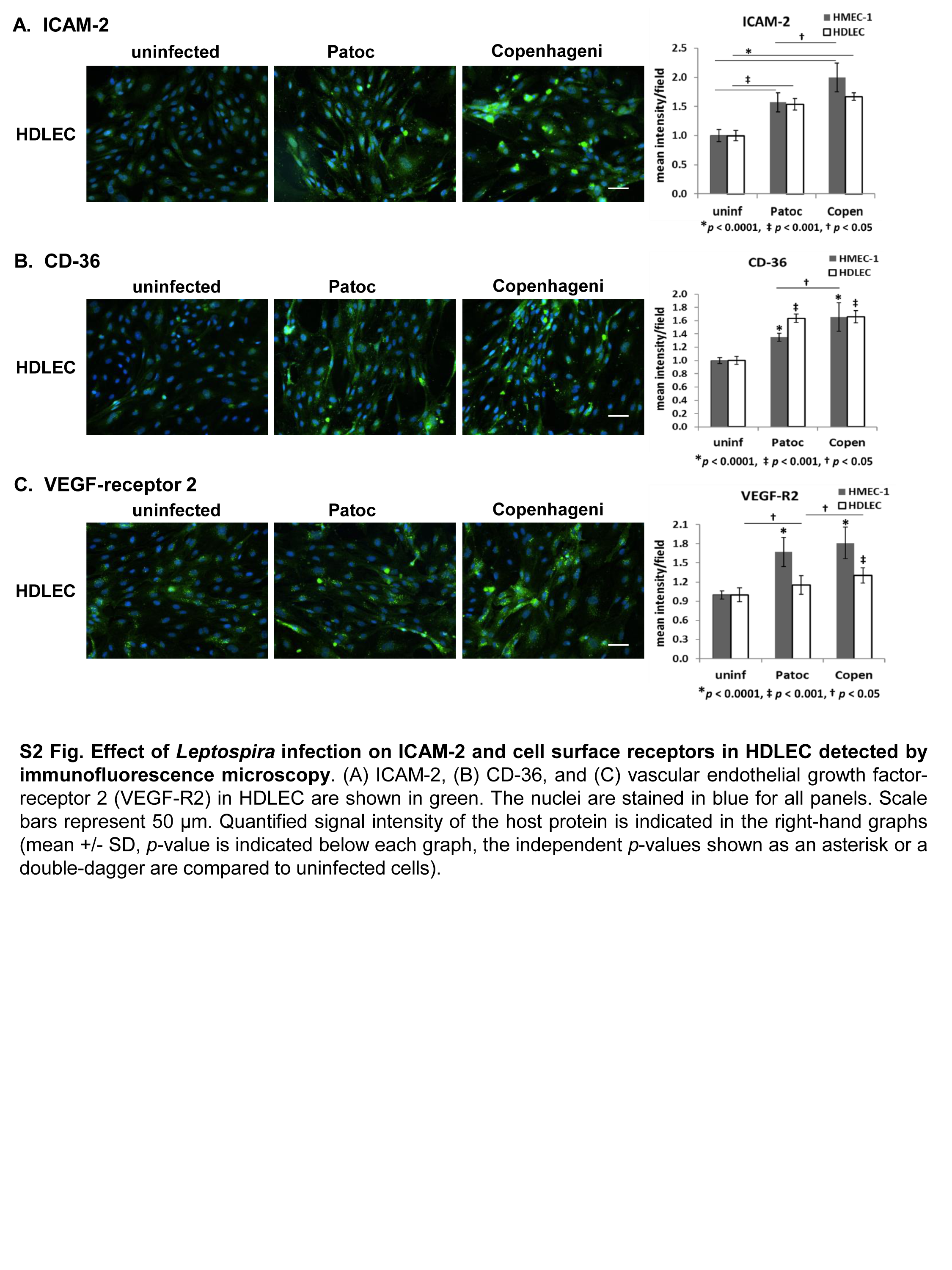

Supplement: S2 Fig — (A) ICAM-2, (B) CD-36, and (C) vascular endothelial growth factor-receptor 2 (VEGF-R2) in HDLEC are shown in green. The nuclei are stained in blue for all panels. Scale bars represent 50 μm. Quantified signal intensity of the host protein is indicated in the right-hand graphs (mean +/- SD, p-value is indicated below each graph, the independent p-values shown as an asterisk or a double-dagger are compared to uninfected cells). (TIF) [file pntd.0005830.s002.tif]

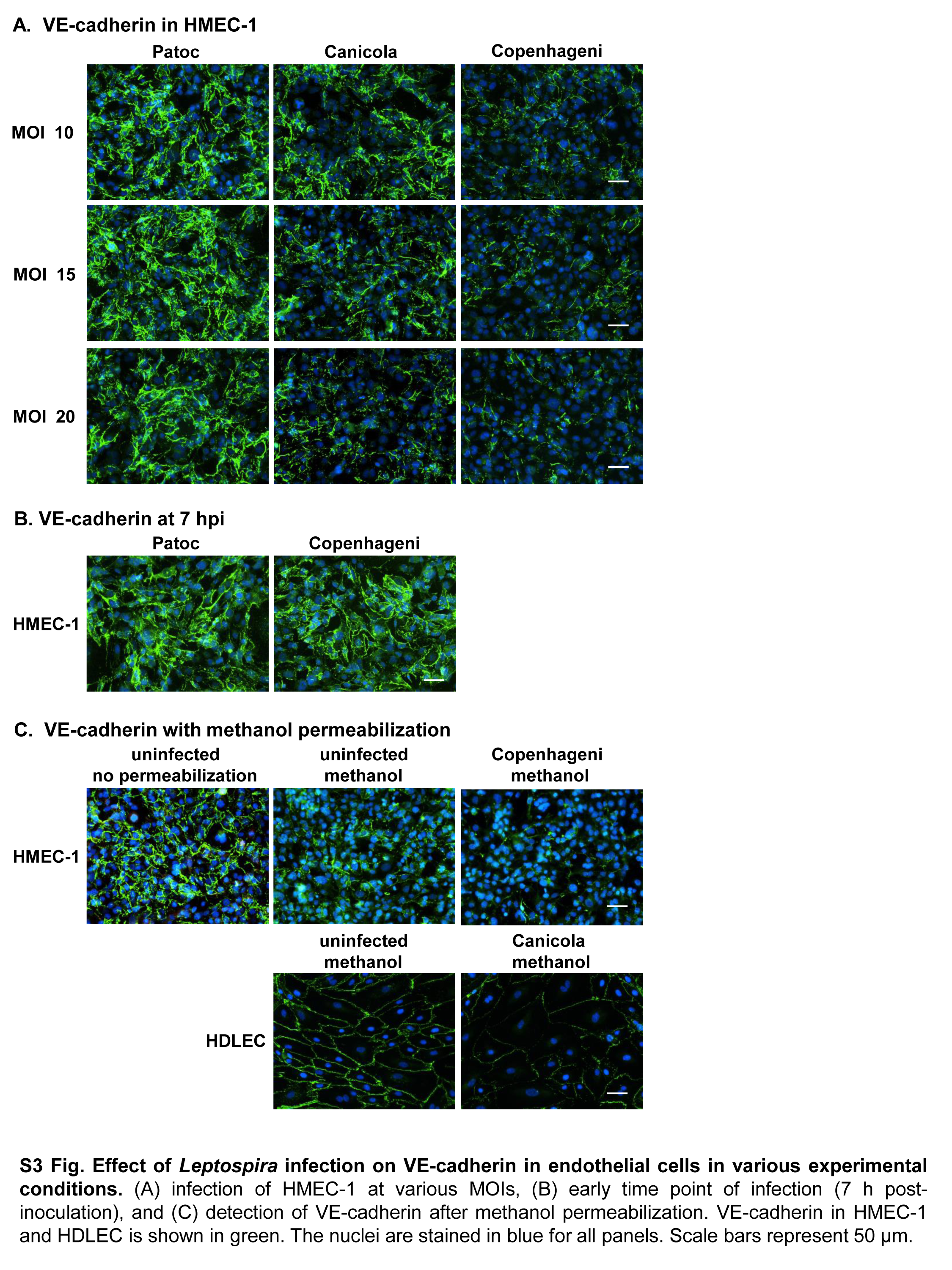

Supplement: S3 Fig — (A) infection of HMEC-1 at various MOIs, (B) early time point of infection (7 h post-inoculation), and (C) detection of VE-cadherin after methanol permeabilization. VE-cadherin in HMEC-1 and HDLEC is shown in green. The nuclei are stained in blue for all panels. Scale bars represent 50 μm. (TIF) [file pntd.0005830.s003.tif]

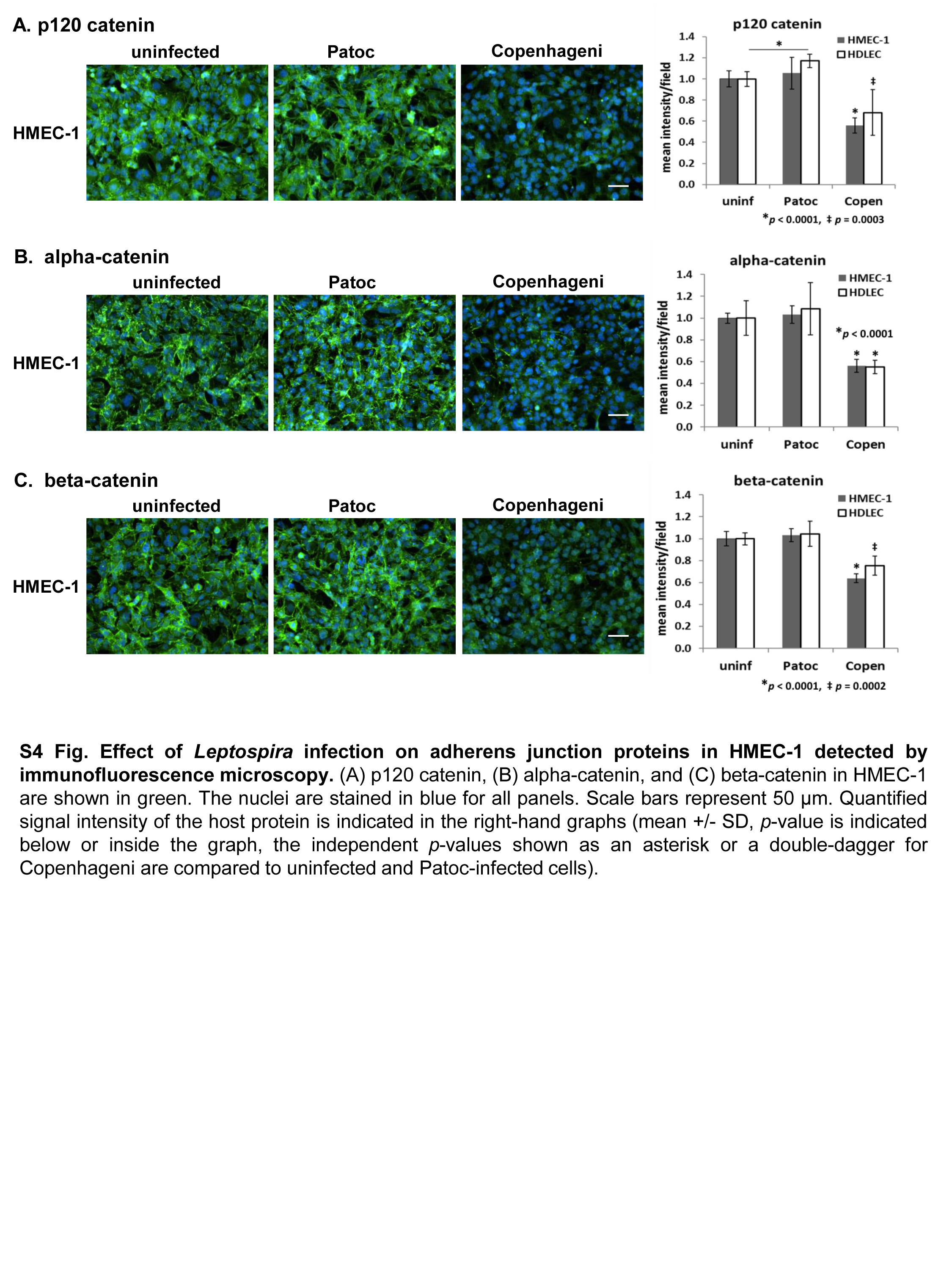

Supplement: S4 Fig — (A) p120 catenin, (B) alpha-catenin, and (C) beta-catenin in HMEC-1 are shown in green. The nuclei are stained in blue for all panels. Scale bars represent 50 μm. Quantified signal intensity of the host protein is indicated in the right-hand graphs (mean +/- SD, p-value is indicated below or inside the graph, the independent p-values shown as an asterisk or a double-dagger for Copenhageni are compared to uninfected and Patoc-infected cells). (TIF) [file pntd.0005830.s004.tif]

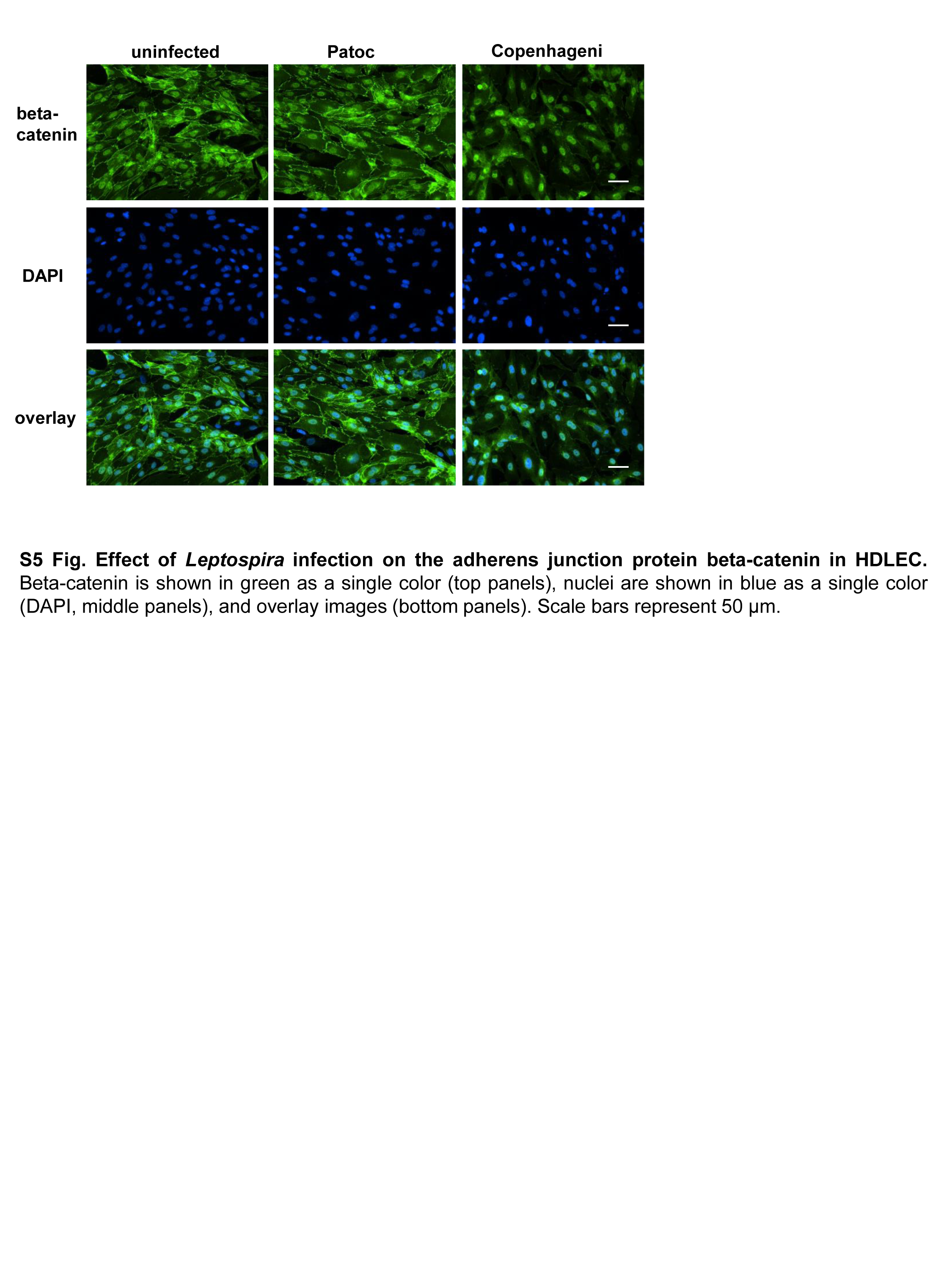

Supplement: S5 Fig — Beta-catenin is shown in green as a single color (top panels), nuclei are shown in blue as a single color (DAPI, middle panels), and overlay images (bottom panels). Scale bars represent 50 μm. (TIF) [file pntd.0005830.s005.tif]

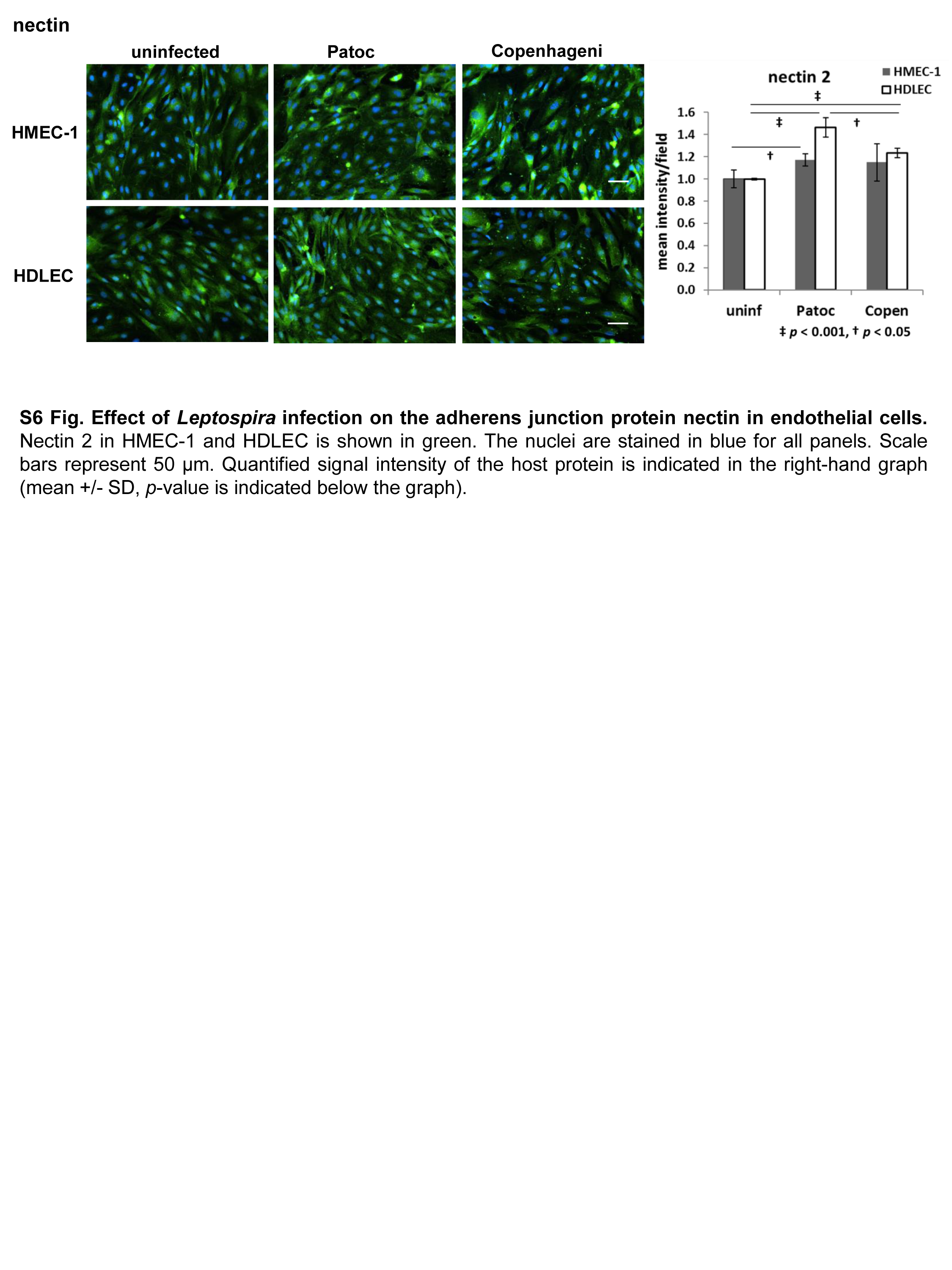

Supplement: S6 Fig — Nectin 2 in HMEC-1 and HDLEC is shown in green. The nuclei are stained in blue for all panels. Scale bars represent 50 μm. Quantified signal intensity of the host protein is indicated in the right-hand graph (mean +/- SD, p-value is indicated below the graph). (TIF) [file pntd.0005830.s006.tif]

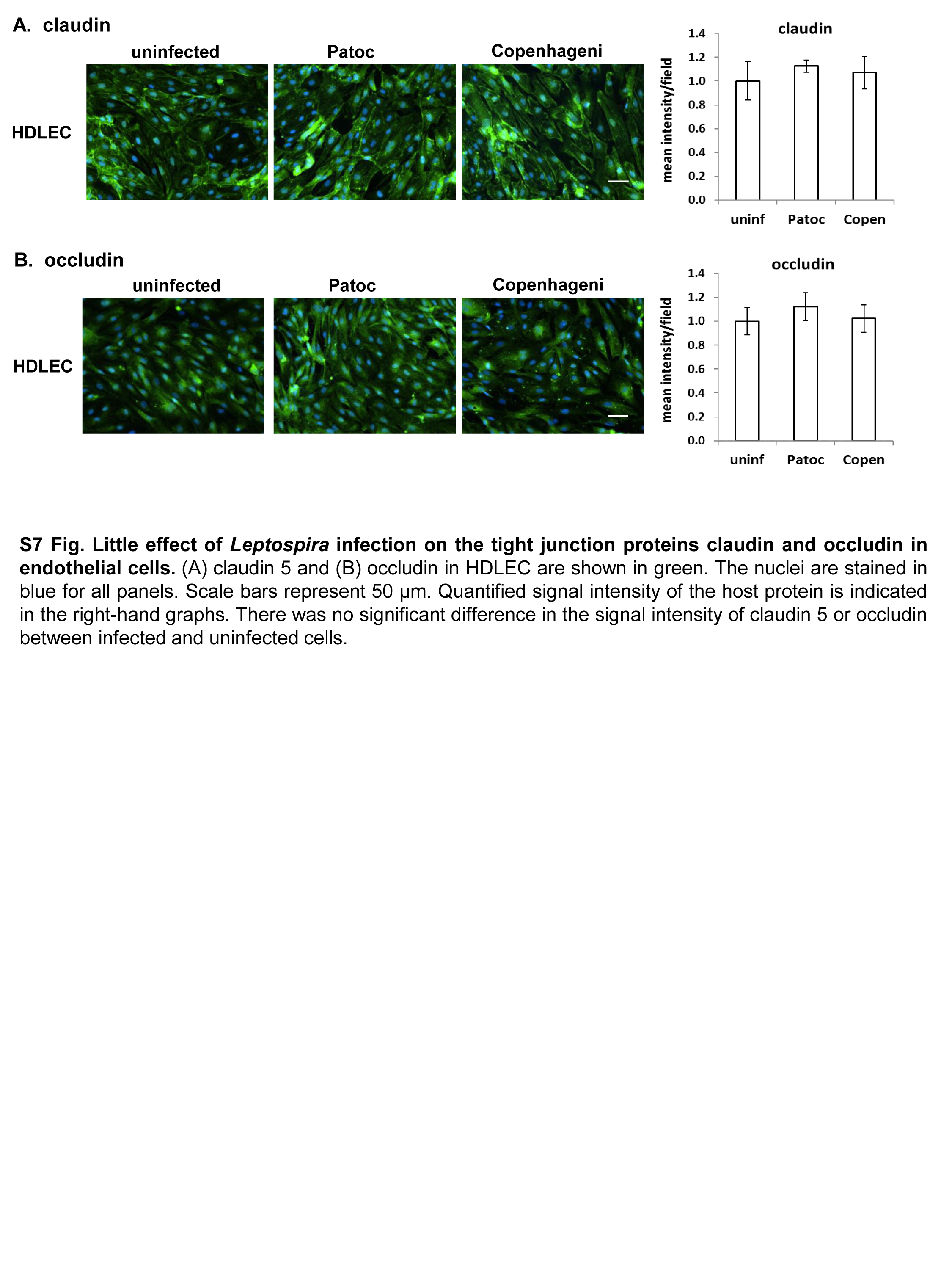

Supplement: S7 Fig — (A) claudin 5 and (B) occludin in HDLEC are shown in green. The nuclei are stained in blue for all panels. Scale bars represent 50 μm. Quantified signal intensity of the host protein is indicated in the right-hand graphs. There was no significant difference in the signal intensity of claudin 5 or occludin between infected and uninfected cells. (TIF) [file pntd.0005830.s007.tif]

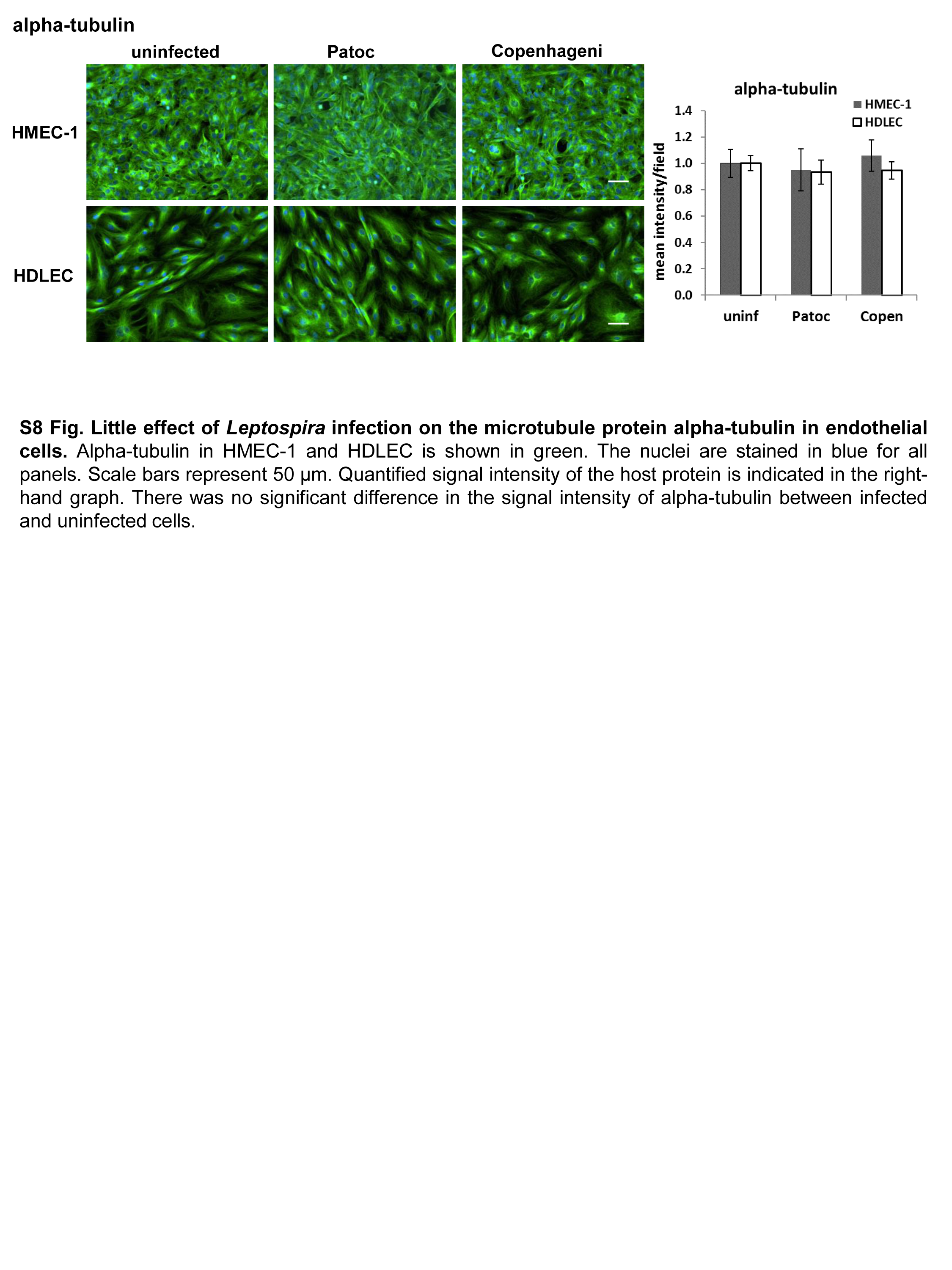

Supplement: S8 Fig — Alpha-tubulin in HMEC-1 and HDLEC is shown in green. The nuclei are stained in blue for all panels. Scale bars represent 50 μm. Quantified signal intensity of the host protein is indicated in the right-hand graph. There was no significant difference in the signal intensity of alpha-tubulin between infected and uninfected cells. (TIF) [file pntd.0005830.s008.tif]

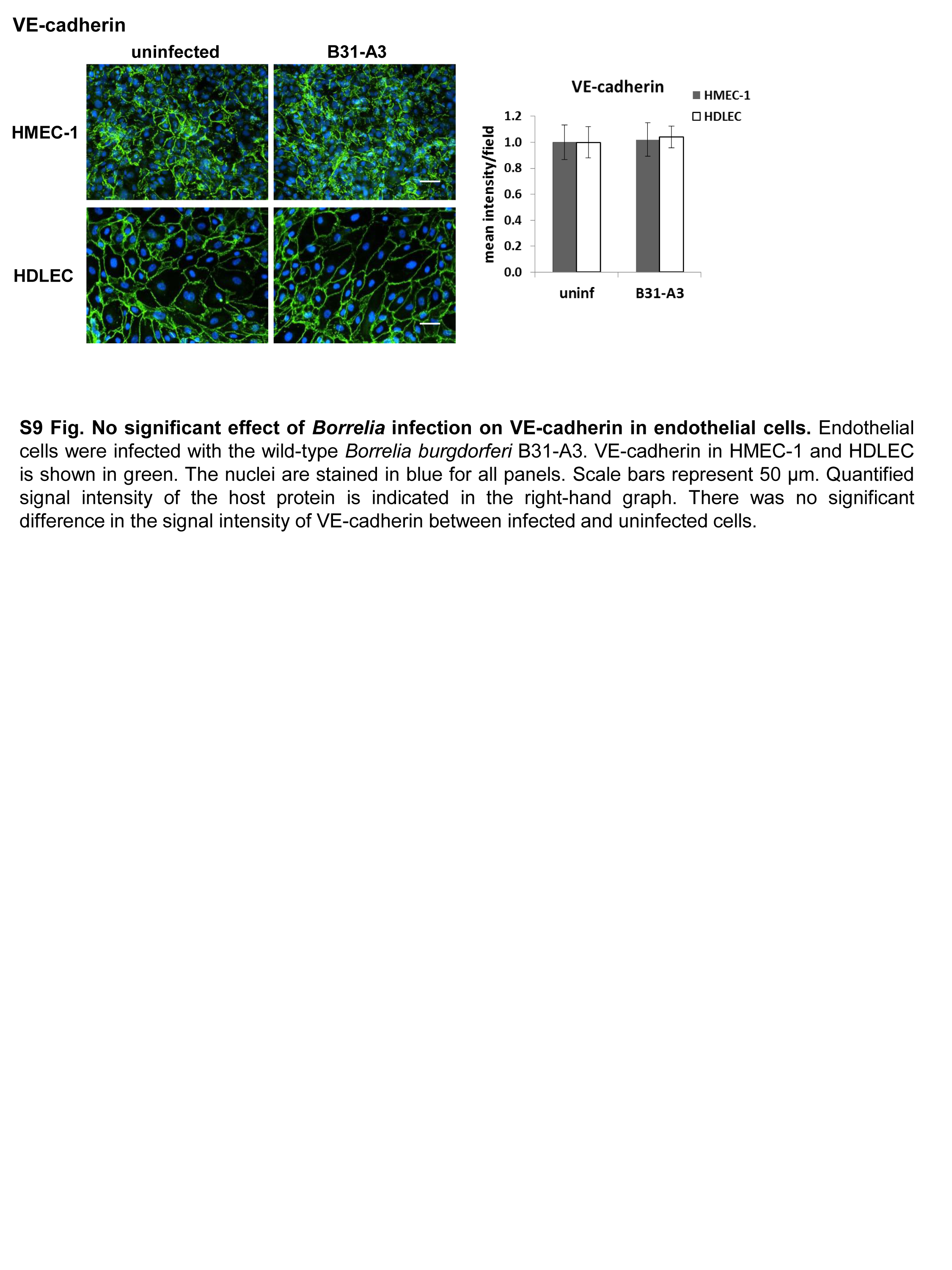

Supplement: S9 Fig — Endothelial cells were infected with the wild-type Borrelia burgdorferi B31-A3. VE-cadherin in HMEC-1 and HDLEC is shown in green. The nuclei are stained in blue for all panels. Scale bars represent 50 μm. Quantified signal intensity of the host protein is indicated in the right-hand graph. There was no significant difference in the signal intensity of VE-cadherin between infected and uninfected cells. (TIF) [file pntd.0005830.s009.tif]
